# Quantitation and Simulation of Single Action Potential-Evoked Ca^2+^ Signals in CA1 Pyramidal Neuron Presynaptic Terminals

**DOI:** 10.1523/ENEURO.0343-19.2019

**Published:** 2019-10-16

**Authors:** Edaeni Hamid, Emily Church, Simon Alford

**Affiliations:** 1 Department of Biological Sciences, University of Illinois at Chicago, Chicago, IL 60607; 2 Department of Anatomy and Cell Biology, University of Illinois at Chicago, Chicago, IL 60612

**Keywords:** calcium buffering, calcium imaging, Monte Carlo simulation, presynaptic, synaptic transmission

## Abstract

Presynaptic Ca^2+^ evokes exocytosis, endocytosis, and synaptic plasticity. However, Ca^2+^ flux and interactions at presynaptic molecular targets are difficult to quantify because fluorescence imaging has limited resolution. In rats of either sex, we measured single varicosity presynaptic Ca^2+^ using Ca^2+^ dyes as buffers, and constructed models of Ca^2+^ dispersal. Action potentials evoked Ca^2+^ transients with little variation when measured with low-affinity dye (peak amplitude 789 ± 39 nM, within 2 ms of stimulation; decay times, 119 ± 10 ms). Endogenous Ca^2+^ buffering capacity, action potential-evoked free [Ca^2+^]_i_, and total Ca^2+^ amounts entering terminals were determined using Ca^2+^ dyes as buffers. These data constrained Monte Carlo (MCell) simulations of Ca^2+^ entry, buffering, and removal. Simulations of experimentally-determined Ca^2+^ fluxes, buffered by simulated calbindin_28K_ well fit data, and were consistent with clustered Ca^2+^ entry followed within 4 ms by diffusion throughout the varicosity. Repetitive stimulation caused free varicosity Ca^2+^ to sum. However, simulated in nanometer domains, its removal by pumps and buffering was negligible, while local diffusion dominated. Thus, Ca^2+^ within tens of nanometers of entry, did not accumulate. A model of synaptotagmin1 (syt1)-Ca^2+^ binding indicates that even with 10 µM free varicosity evoked Ca^2+^, syt1 must be within tens of nanometers of channels to ensure occupation of all its Ca^2+^-binding sites. Repetitive stimulation, evoking short-term synaptic enhancement, does not modify probabilities of Ca^2+^ fully occupying syt1’s C2 domains, suggesting that enhancement is not mediated by Ca^2+^-syt1 interactions. We conclude that at spatiotemporal scales of fusion machines, Ca^2+^ necessary for their activation is diffusion dominated.

## Significance Statement

Presynaptic Ca^2+^ influx, buffering and removal is essential to evoke synaptic transmission and therefore fundamental to nervous system function. It is also responsible for many forms of synaptic plasticity. Thus, it is important to understand fluxes of Ca^2+^ at the scale of the molecules with which it interacts. However, physical limitations prevent its imaging at such temporo-spatial scales. By combining a quantitative approach using Ca^2+^ dyes as buffers to determine characteristics of Ca^2+^ entry, buffering and removal from presynaptic varicosities, with simulation of Ca^2+^, its binding partners in and removal from varicosities, we provide a detailed quantitative analysis of Ca^2+^ at molecular scales. This enables insights into its molecular targets, and its effects on SNARE complexes and interacting proteins.

## Introduction

Presynaptic Ca^2+^ entry through voltage-gated Ca^2+^ channels (VGCCs) causes exocytosis (Katz and Miledi, 1967). Exocytosis may require just one VGCC (Stanley, 1993; Bucurenciu et al., 2010; Weber et al., 2010; Eggermann et al., 2012; Scimemi and Diamond, 2012) or their clustering at microdomains (Llinás et al., 1992a; Shahrezaei and Delaney, 2004; Oheim et al., 2006), and their numbers may vary at active zones (Lübbert et al., 2019). Additionally, Ca^2+^ plays other roles in presynaptic terminals including modifying repetitive neurotransmission (Delaney et al., 1989; Zucker and Regehr, 2002), and receptor-mediated neuromodulation (Yoon et al., 2007; Gerachshenko et al., 2009).

Presynaptic Ca^2+^ transients are resolvable with Ca^2+^ dyes (DiGregorio and Vergara, 1997; Cochilla and Alford, 1998; Koester and Sakmann, 2000) at millisecond times and over the size of the entire terminal. However, Ca^2+^ is used too locally and rapidly to be imaged within the dimensions of Ca^2+^-binding molecules that cause exocytosis (Adler et al., 1991; Sabatini and Regehr, 1996). Nevertheless, dyes can quantify presynaptic Ca^2+^ entry, buffering and removal (Neher and Augustine, 1992; Koester and Sakmann, 2000; Jackson and Redman, 2003; Brenowitz and Regehr, 2007). This approach requires calibration of dye concentrations, fluorescence, and Ca^2+^-binding properties within cells.

Neuronal Ca^2+^ binding requires various intracellular proteins. Bulk binding is dominated by EF-hand Ca^2+^-binding proteins (Burgoyne, 2004), but also occurs at C2 domains on numerous signaling proteins, including PKC (Farah and Sossin, 2012) and synaptotagmin1 (syt1; Chapman, 2002). CA1 pyramidal neuron somata and dendrites contain ∼40 µM of the Ca^2+^-binding protein calbindin_28K_ (Baimbridge et al., 1992; Müller et al., 2005), which may represent their dominant buffer (Yi, 2013), although other buffers are present. Indeed, calmodulin is present at presynaptic terminals (Llinás et al., 1991; Hinds et al., 2003). Buffering characteristics of these EF-hand proteins have been characterized *in vitro* (Nägerl et al., 2000; Faas and Mody, 2012) and by modeling *in situ* (Schmidt et al., 2005), allowing their impact on local Ca^2+^ signaling to be modeled.

Syt1 is widely considered the principal Ca^2+^ sensor for evoked release in pyramidal synapses (Geppert et al., 1994). In many synapses, exocytosis requires close association (<100 nm) between Ca^2+^ entry and synaptotagmin (Adler et al., 1991; Martens et al., 2007; Chapman, 2008; Südhof and Rizo, 2011). Ca^2+^ buffers modify Ca^2+^ diffusion, and Ca^2+^-synaptotagmin interactions. Although widely accepted (DiGregorio and Vergara, 1997; Koester and Sakmann, 2000) this is poorly characterized in presynaptic terminals. Syt1 has two Ca^2+^-binding domains (C2A and C2B; Perin et al., 1991), which bind three and two Ca^2+^ ions and is the Ca^2+^ sensor in CA1 axons. However, it is unclear whether all syt1 Ca^2+^ sites must bind Ca^2+^ given very low affinities for the C2B domain. Indeed, only one domain is necessary for membrane interaction (Davletov and Südhof, 1993), although this low affinity increases on association with lipid membranes containing phosphatidylinositol 4,5-bisphosphate PI(4,5)P_2_ (Radhakrishnan et al., 2009; van den Bogaart et al., 2012).

To understand how much presynaptic Ca^2+^ enters presynaptic terminals, how it interacts with presynaptic Ca^2+^ buffers and with fusogenic targets such as syt1, we have quantified [Ca^2+^]_i_ in CA1 presynaptic terminals during action potentials. This allowed us to simulate action-potential-evoked Ca^2+^ entry, binding, buffering and dispersal at individual terminals using Monte Carlo (MCell) simulation (Kerr et al., 2008) and investigate its interaction with syt1 at resolutions that evoke exocytosis. Combining our results from quantitative analysis of exogenous dye/buffers and computational modeling demonstrate the complex impacts of temporo-spatial scales on Ca^2+^ diffusion Ca^2+^ buffering, buffer saturation during evoked presynaptic Ca^2+^ entry.

## Materials and Methods

### The preparation

Experiments were performed on hippocampal slices (300 µm) of male or female 20- to 22-d-old Sprague Dawley rats anesthetized with isoflurane and decapitated. Hippocampi were isolated under semi-frozen Krebs–Henseleit solution: 124 mM NaCl, 26 mM NaHCO_3_, 1.25 mM NaH_2_PO_4_, 3 mM KCl, 2 mM CaCl_2_, 1 mM MgCl_2_, and 10 mM D-glucose, bubbled with 95% O_2_-5% CO_2_, sliced using a Vibratome. The recording chamber was superfused at 2 ml/min and maintained at 28 ± 2°C. Experiments were performed in accordance with institutional guidelines of the University of Illinois at Chicago and the Association for Assessment and Accreditation of Laboratory Animal Care.

### Electrophysiology

**Figure 1.**
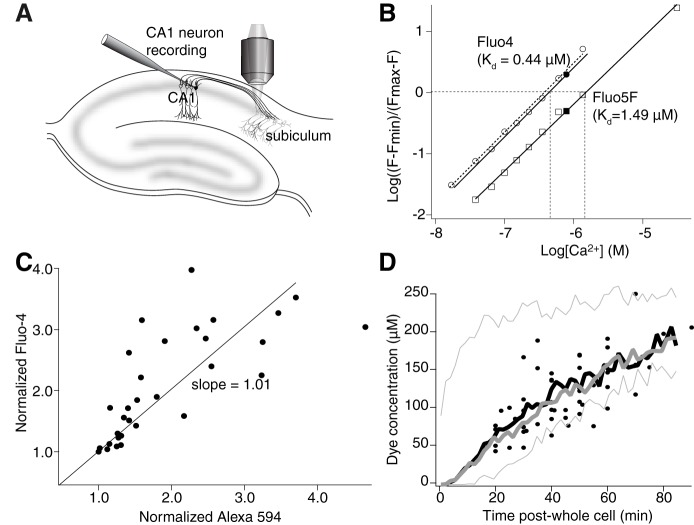
Calibration of dye diffusion and Ca^2+^ response. ***A***, Recording arrangement for imaging presynaptic varicosities. CA1 pyramidal neurons were whole-cell patch-clamped with electrodes containing Fluo-4 (or 5F) and Alexa Fluor 594 hydrazide. Axons and varicosities were traced to the subiculum by imaging Alexa Fluor 594 hydrazide and Ca^2+^ was imaged using the Fluo dye. ***B***, Calibration of the Ca^2+^-sensitive dyes. Fluo-4 (circles) and Fluo-5F (squares) were imaged on the confocal system used for all measurements. Fluorescence intensity was measured over a range of Ca^2+^ standard concentrations in fixed concentrations of EGTA. Log-log plots gave a slope (Hill coefficient) of 1. Ca^2+^ concentrations were applied to a patch-clamped cell by application of ionomycin. From saturated Ca^2+^ concentrations, zero Ca^2+^ and a fixed value of Ca^2+^ (0.78 µM in EGTA-buffered Ca^2+^ solution), the closed circle (Fluo-4) and closed square (Fluo-5F) values in the graphs were calculated. These values were used to correct the plots to values in the intracellular environment (solid lines) and to calculate values of *K*_d_ for the two dyes in the cells (0.44 and 1.49 µM). ***C***, Dyes introduced from the patch pipette showed equal diffusion rates into axon varicosities. Intensities of Fluo-4 and Alexa Fluor 594 hydrazide measured by separate illumination at 488 and 568 nm, respectively, were normalized to the value of the fluorescence obtained following identification of a varicosity (20 min after whole-cell access) and plotted against one another for points measured over the next 20 min. The slope of a line fitted to this data (through 0,0, because both dye concentrations were zero at the experiment start) is close to unity. ***D***, Fluorescence intensities of varicosities from all neurons in which Alexa Fluor 594 hydrazide was loaded with Fluo-4 (11 neurons; filled black circles). Overlaid on this data are results of simulations of diffusion of modeled Fluo-4 molecules (mol wt 736 g mol^−1^, thick gray line) and Alexa Fluor 594 (mol wt 737 g mol^−1^; thick black line). These were detected 250 µm from the somata. The thin gray lines indicate the Alexa Fluor 584 concentrations simulated closest to the soma (70 µm) and at the extreme range of distances (600 µm from the soma).

CA1 pyramidal neurons were whole-cell clamped following visual identification using an upright microscope with an Axopatch 200A amplifier (Molecular Devices). Patch pipettes (4–5 MΩ) contained the following: 146 mM potassium methane sulphonate, 2 mM MgCl_2_, 0.025 mM EGTA, 9.1 mM HEPES, 5 mM ATP, and 2.5 mM GTP, pH adjusted to 7.2 with KOH. Pipettes were also filled with either Fluo-4 (1 mM) or Fluo-5F (200 µM) and Alexa Fluor 594 hydrazide (250 µM). Subicular pyramidal neurons were recorded under whole-cell conditions but were held under voltage clamp to record synaptic inputs. In these latter neurons access resistance was monitored with a 10 mV voltage step before each episode. Focal stimuli (0.2 ms, 20 µA or less) were applied over CA1 axons using glass-insulated monopolar tungsten microelectrodes. Cells were labeled with dye by allowing sufficient time for diffusion from the patch pipette in the live cell. Axons were tracked from the soma to their presynaptic terminals in the subiculum (Fig. 1*A*; Hamid et al., 2014).

### Imaging

Confocal microscopy was used to image individual varicosities of CA1 pyramidal neurons, with a 60 × 1.1 NA water-immersion lens using a modified Bio-Rad MRC 600 confocal microscope with excitation wavelengths at 488 and 568 nm (Bleckert et al., 2012). Ca^2+^-sensitive dyes of one of two different affinities to Ca^2+^ were visualized in each experiment. Dye concentration was determined by pairing these dyes with a Ca^2+^-insensitive dye (Alexa Fluor 594 hydrazide, molecular weight, 736 g mol^−1^, identical to Fluo-5F and almost identical to that of Fluo-4, 737 g mol^−1^). Co-diffusion of the Fluo-4 and Alexa Fluor 594 hydrazide was demonstrated by recording absolute values of the fluorescence at axonal varicosities at rest over time. Alexa Fluor 594 hydrazide was excited with a 568-nm laser and imaged in longpass (>580 nM). Fluo dyes were separately excited with a 488-nm laser and imaged in bandpass (510–560 nm). Images were taken separately to ensure no cross channel bleed-through.

Ca^2+^-binding properties of Fluo-4 and Fluo-5F were determined using Ca^2+^ standards (Invitrogen) at 28 ± 2°C (the temperature at which experiments were performed) and pH 7.2 (to which the intracellular patch solutions were buffered). Log plots of these data points were used to determine *K*_d_ (Fig. 1*B*). Calibrated measurements of dye fluorescence within neurons were also made for each dye (*n* = 2 for Fluo-5F and *n* = 2 for Fluo-4) using whole-cell recordings obtained when the patch electrode contained either Fluo-4 or Fluo-5F. Whole-cell access was maintained until soma and dendrites were clearly labeled. The electrode was then carefully withdrawn. Baseline fluorescence intensities were measured. The Ca^2+^ ionophore, ionomycin (5 µM) was added to the superfusate. Fluorescence intensities were measured at 2-min intervals until the signal reached a stable maximum. The superfusate was then replaced with a solution containing 0 Ca^2+^ and 10 mM EGTA and images taken until the fluorescence intensity reached a stable minimum. This solution was then replaced with a solution containing buffered Ca^2+^-EGTA ([Ca^2+^] = 0.78 µM) and fluorescence in the neuron was measured. Ratios of maxima to minima were very close to those obtained with standards. The standard data points were then plotted over the log plots obtained *in vitro*. A small correction was applied to the calculated *K*_d_ for Fluo-4 (Fig. 1*B*, black line) but the data obtained with Fluo-5F gave a value of *K*_d_ that was not measurably different from that obtained *in vitro*. These values (Fluo-4 kDa = 0.44 µM, F_min_/F_max_ = 0.066 and Fluo-5F *K*_d_ = 1.49 µM, F_min_/F_max_ = 0.023) were used in all subsequent calculations.

To determine whether Ca^2+^ dyes and Alexa Fluor dyes diffused at similar rates, Fluo-4 and Alexa Fluor 594 hydrazide signals at varicosities were identified within 20 min of whole-cell access in 10 neurons. Signal strength was normalized to this time point for both dyes. A comparison of the increase in dye intensity over the following 20 min with no stimulation reveals strong correlation with a slope of 1.01 (Fig. 1*C*, this fit was forced through the origin because both dyes must be at a concentration of zero at the start of the experiment. Without this, the slope was 0.94).

As an independent control to confirm that the slight difference in molecular weights alone does not alter rates of diffusion of the two dyes in the axon, experimentally measured diffusion of Alexa Fluor 594 hydrazide was compared to diffusion of dye in a MCell simulation. Fluorescence intensities of varicosities from all recorded neurons in which Alexa Fluor 594 hydrazide was co-loaded with Fluo-4 (11 neurons; filled black circles) were measured (Fig. 1*D*). Overlaid on this data are results of simulations (MCell; Kerr et al., 2008) of diffusion of modeled dyes. For these simulations a 3D mesh model cell was created in which a spherical volume (10 µm diameter) contained molecules with diffusion constants to simulate Fluo-4 (mol wt 736 g mol^−1^) and Alexa Fluor 594 hydrazide (mol wt 737 g mol^−1^). These concentrations were kept constant at this site. Contiguous with this site was a cylindrical mesh of diameter 0.12 µm to simulate the axon diameter, interspersed with 1-µm diameter varicosities at 4-µm intervals. The axon diameter was derived from two sources. (1) Imaging allowed the ratio of total varicosity intensity to be compared with axon intensity. Thus, relative diameter of the axons and varicosities can be calculated from the ratios of the fluorescence intensities (varicosity/axon = 8.3) and the measured diameters of the varicosities (1 µm) assuming dye concentrations are the same in both compartments. The resultant ratio allowed calculation of the axon diameter as a ratio of the varicosity diameter. (2) This result agreed closely with electron microscopy of pyramidal cell axons (Harris and Weinberg, 2012). Resultant simulated dye concentrations were calculated at a distance of 250 µm from the somata (Fig. 1*D*, thick black and gray lines). This represents the median range of the distance of experimentally imaged varicosities. The thin gray lines indicate the Alexa Fluor 584 concentrations simulated in varicosities closest to the soma (70 µm; top) or furthest from the soma (600 µm from the soma: bottom).

Note that the small difference in diffusion rate simulated between Alexa Fluor 594 (Fig. 1*D*, gray) and Fluo-4 (Fig. 1*D*, black) made no measurable difference to the simulated dye diffusion rates. Additionally, the accumulation of these simulated dyes was overlapping with the experimentally measured fluorescence increase of Alexa Fluor 594 hydrazide (black circles) indicating that the rate of rise of dye concentration in the varicosities is consistent with simple diffusion. Thus, both dyes reach the varicosity at the same rate, which allows the use of Alexa Fluor 594 hydrazide as a “standard candle” for measuring dye concentration. Thus, Alexa Fluor 594 hydrazide fluorescence in axon varicosities was used to determine the concentration of Ca^2+^-sensitive dye in the terminal by calculation of that fluorescence as a fraction of its fluorescence in the recording pipette where its concentration was known.

The fluorescence intensity of Alexa Fluor 594 hydrazide fluorescence was measured throughout the experiment in the terminal using fixed parameters on the imaging system. A plot of intensity against time approached an asymptote toward 60 min after obtaining whole-cell access. The absolute fluorescence at the electrode tip was compared to that of the axon varicosities. At the end of the experiment the axon typically blebbed to ∼5 µm, large enough for the microscope point spread function to allow for absolute fluorescence of the axon to be measured. This phenomenon was never present during the recording of stimulation-evoked Ca^2+^ transients. Its occurrence was observed subsequent to a rise in resting Ca^2+^ seen after about an hour of recording. Fluorescence in the tip of the pipette where the dye concentration was known was measured in the tissue at the same depth as the axon. This allowed calculation of the axon dye concentration after the experiment ended. It was then straightforward to compare all previously measured values of Alexa Fluor 594 fluorescence, to give absolute dye concentrations throughout the experiment. Recordings in which all of these criteria could not be met were rejected from analysis. Absolute Ca^2+^ concentrations were calculated in each varicosity using Equations 1, 2 (below). For these calculations we obtained saturated Fluo-4 or Fluo-5F intensity values in varicosities at the end of the experiment by repetitive stimulation and calculated minimum fluorescence values determined from the data in Figure 1*B*.

### Effects of introduction of buffers to cell compartments

It is possible to calculate the intracellular Ca^2+^ concentration ([Ca^2+^]_i_). For a non-ratiometric dye with a Hill coefficient of 1, [Ca^2+^]_i_ is determined from Equation 1:

(1) $${\left[Ca^{2+}\right]}_{i}=K_{d}\frac{\left(F-F_{min}\right)}{\left(F_{max}-F\right)}.$$

The Ca^2+^ dye minimum fluorescence intensity (F_min_) was calculated as a ratio of F_max_ determined from the dye calibration results (Fig. 1*B*), and from each cell at the end of the experiment. Absolute values of F_min_ and F_max_ were corrected by the observed value of Alexa Fluor 594 for each time point as a ratio of its value at the end of the experiment when F_max_ was measured. A corrected value of Ca^2+^ dye fluorescence in the varicosity (F) was calculated from the measured varicosity fluorescence (F_meas_) at each time point used for analysis and then re-expressed as a ratio of F_max_, corrected by comparison to the Alexa Fluor 594 signal throughout the experiments. This is because F_max_ was determined at the end of the experiment and consequently needed to be scaled for each time point at which measurements were made during the experiment. Thus, F is given by:

(2) $$F=\frac{D_{F}}{D}.\frac{F_{meas}}{F_{max}},$$

where *D* is the Alexa Fluor 594 fluorescence at each time, and *D_F_* is the final Alexa Fluor 594 fluorescence. Thus, for experiments using Fluo-4 or Fluo-5F, where F and F_min_ in Equation 1 are ratios of F_max_, and F_max_ = 1, [Ca^2+^]_I_ was calculated as follows:

(3) $${\left[Ca^{2+}\right]}_{i}=0.44\frac{\left(\frac{D_{f}.F_{meas}}{D}-0.023\right)}{\left(1-\frac{D_{f}.F_{meas}}{D}\right)}.$$

These experiments required constant laser intensity and recording parameters throughout the experiment. To minimize photobleaching, imaging was performed only transiently during evoked responses (1 s per stimulus, <15 s total per experiment). To use calcium-sensitive dyes as buffers to investigate the fate of Ca^2+^ that enters presynaptic terminals on stimulation, we must calculate their buffering capacities (κ_dye_) in the cytosol of the terminal. Since each molecule of dye binds just one Ca^2+^ ion, the Hill equation with a coefficient of one can be used to calculate κ_dye_ over a change in Ca^2+^ concentration (Δ[Ca^2+^]_i_) from [Ca^2+^]_1_ to [Ca^2+^]_2_.

(4) $$\kappa_{dye}=\frac{\Delta \left[Ca_{dye}\right]}{\Delta {\left[Ca^{2+}\right]}_{i}}=\frac{\left[Dye_{total}\right]}{k_{d}\left[\left(1+\frac{{\left[Ca^{2+}\right]}_{2}}{k_{d}}\right)\left(1+\frac{{\left[Ca^{2+}\right]}_{1}}{k_{d}}\right)\right]},$$

where [Ca_Dye_] is the concentration of Ca^2+^-bound dye, and [*Dye_total_*] is the total dye concentration. Note that this approach takes into account the change in [Ca^2+^]_i_ in the pyramidal cell terminals during the stimulus which is large (approximately 1 µM). Other approaches with smaller Ca^2+^ changes use resting [Ca^2+^]_i_ as a basis for calculating κ_dye_ (Neher and Augustine, 1992).

When a rapid Ca^2+^ pulse enters a cell compartment, free Ca^2+^ may be removed first by binding to intracellular endogenous buffers, and possibly by diffusion into neighboring compartments, and then by pumps. We have used methods used by Jackson and Redman (2003) originally described by Melzer et al. (1986) to determine the buffering characteristics of Ca^2+^ in CA1 pyramidal neuron presynaptic varicosities. From these methods we can determine the quantity of calcium entering the varicosity, the mean free [Ca^2+^]_i_ within the varicosity immediately after the stimulus, the endogenous Ca^2+^ buffering capacity, and the rate of removal of Ca^2+^ from the cytosol. From this we have developed simulations of Ca^2+^ entry, diffusion, and buffering in the presynaptic terminal.

The relationship between Ca^2+^ unbinding rates from the dye and rebinding either to dye or endogenous buffers can be used to calculate endogenous buffering capacity (κ_end_) of the terminal. If the value of κ_dye_ varies during the experiment then we assume a constant rate of Ca^2+^ extrusion from the terminal (τ_ext_). The decay rate (τ) of a pulse-like Ca^2+^ signal in a cell compartment is described by the equation:

(5) $$\tau =\tau_{ext}(1+\kappa_{end}+\kappa_{dye}).$$

Thus, we obtained values of κ_end_ by fitting Equation 5 to plots of τ from experimental data vs κ_dye_ from Equation 4. This approach does have drawbacks, if processes modifying Ca^2+^ removal or adding to cytosolic Ca^2+^ occur after action potentials. Such processes include diffusion of the Ca^2+^-dye complex from the measured compartment, or release of Ca^2+^ from internal stores. In Equation 5, these effects are grouped into a single variable κ_end_. Nevertheless, the result (κ_end_) can be obtained independently of computed absolute values of Ca^2+^, or even of background fluorescence measurement errors. It therefore serves as an independent measure of whether the alternative following measurements of κ_end_ are reasonable.

By measuring the peak amplitude of the free Ca^2+^ transient throughout the varicosity over a range of values of κ_dye_, we may assume that the total change in Ca^2+^ concentration due to a stimulus is described by the equation:

(6) $$\Delta {\left[Ca\right]}_{total}=\Delta {\left[Ca^{2+}\right]}_{i}+\Delta \left[Ca_{dye}\right]+\Delta [Ca_{end}],$$

(7) $$\Delta \left[Ca_{dye}\right]=\kappa_{dye}•\Delta {\left[Ca^{2+}\right]}_{i},$$

and

(8) $$\Delta \left[Ca_{end}\right]=\kappa_{end}•\Delta {\left[Ca^{2+}\right]}_{i},$$

where *[Ca_end_]* is the concentration of endogenous buffer bound to Ca^2+^ and *Δ[Ca]_total_* is the total stimulus-evoked change in calcium concentration in the cell compartment. Thus, combining these equations, we may state:

(9) $$\Delta {\left[Ca^{2+}\right]}_{i}=\frac{\Delta {\left[Ca\right]}_{total}}{\left(1+\kappa_{dye}+\kappa_{end}\right)},$$

where Δ[Ca^2+^]_i_ varies with κ_dye_. Values of Δ[Ca^2+^]_i_ are computed from our data using Equation 3 and values of κ_dye_ from Equation 4. For each action potential, as the value of κ_dye_ rises it will come to dominate binding of Ca^2+^ entering the cell compartment. This approach is useful because the value of Δ[Ca_dye_] can be calculated for each action potential in each presynaptic terminal as the value of κ_dye_ increases by diffusion of dye from the soma. Equations 6–8 can also give:

(10) $$\Delta {\left[Ca\right]}_{total}=\Delta \left[Ca_{dye}\right].\left\{\frac{\left(\kappa_{end}+1\right)}{\kappa_{dye}}+1\right\}.$$

Values of Δ[Ca]_total_, and κ_end_ can be determined by extracting constants from fits of either Equation 9 or Equation 10. The true value of peak Δ[Ca^2+^]_I_ in the varicosity when no dye is present is obtained by extrapolating the fit to the y intercept in Equation 9, where κ_dye_ = 0. To calculate total Ca^2+^ entering the terminal from the concentrations obtained from either Equation 9 or Equation 10, we calculated varicosity volumes from images using Alexa Fluor 594 hydrazide. Varicosities are larger than the smallest structures that can be imaged in our microscope. Point spread data from 0.2-µm latex microspheres were determined by imaging under the same light path as all data in this study (568-nm excitation, longpass emission). The point spread was Gaussian in *x*-*y* and *z* dimensions with an *x*-*y* dimension half maximal width of 0.45 µm.

Varicosities approximated ellipsoids with the long axis along the line of the axon. We measured length (l) and width (w), assuming depth was similar to the width because it was not possible to obtain sufficient z-plane resolution to accurately determine depth. Mean measured varicosity length (l) was 2.3 ± 0.2 µm and width (w) was 1.2 ± 0.1 µm. These values are quite similar to terminal sizes obtained from electron microscopic images (Harris and Weinberg, 2012).

Assuming the varicosities were ellipsoid, volume of the varicosity is given by:

(11) $$volume=\frac{4}{3}\pi \left\{{\left(\frac{w}{2}\right)}^{2}.\frac{l}{2}\right\}.$$

Chemicals were obtained as follows: Alexa Fluor dyes, Fluo-4, Fluo-5F, and Ca^2+^ standards from Thermo Fisher; salts, buffers, etc. from Sigma.

### Simulations

MCell simulations were applied to Ca^2+^ buffering within a model of the CA1 axon varicosity based on the data obtained experimentally in this study. Simulations were run in the MCell environment (Kerr et al., 2008) in which a 3D mesh model of the presynaptic terminal was created based on measurements determined from these experiments and from electron microscopic images of hippocampal presynaptic varicosities (Harris and Weinberg, 2012). Ca^2+^ entry, diffusion binding and removal from the terminal were modeled using initial parameters obtained from experimental data in this study, and from the literature and from published sources. Possible Ca^2+^-binding proteins and their concentrations were investigated by comparing multiple parameters from experimental data and the results of simulations. All of the parameters used are outlined in Tables 1–3. An animated visualization of these simulations are viewable: https://anatomy.uic.edu/faculty/index.html?fac=simontalford&cat=all. Similarly, MCell parameter sets are available on this website.

### Statistics

Data were combined from numerous recordings from a total of 18 neurons to calculate values indicated in tabular form. Fits to datasets over for a number of equations were performed in Igor Pro (Wavemetrics). Errors intervals from fitted data represent the 90% confidence limits of those fits. To compare simulation results to experimental data, results were considered to validate the model if these simulation datasets fell within the 95% confidence intervals of the experimental data. In fact, for each of these validations, simulations which fell within the 90% confidence interval indicated a stronger correlation. Otherwise errors are reported as the SEM. Significance was tested with two-tailed Student’s *t* test or two-factor ANOVAs where appropriate. We used an α level of 0.05 for significance for statistical tests.

## Results

### Resting Ca^2+^ concentrations in axonal varicosities

To measure presynaptic Ca^2+^, Ca^2+^ sensitive and insensitive dyes were introduced to CA1 pyramidal neurons from somatic whole-cell pipettes containing Ca^2+^-sensitive dye, and Alexa Fluor 594 hydrazide (250 µM). After 20 min, the axon was traced by imaging Alexa Fluor 594 (Hamid et al., 2014). We initially used Fluo-4 (1 mM) as a Ca^2+^ sensor. Alexa Fluor 594 fluorescence was used to measure dye concentration. We assume co-diffusion of the dyes which have similar molecular weights. Thus, Ca^2+^ dye concentrations were calculated throughout each experiment (Materials and Methods) using Alexa Fluor 594 hydrazide as a standard.

**Figure 2.**
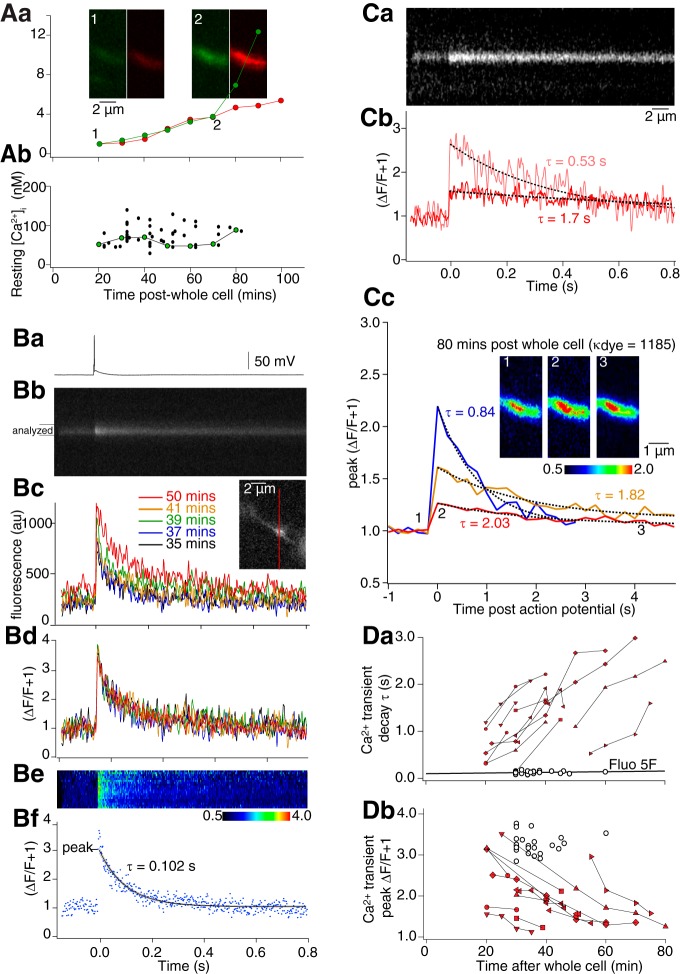
Effects of high- and low-affinity dyes on presynaptic Ca^2+^ transients. ***Aa***, CA1 pyramidal neurons whole-cell recorded and filled with Alexa Fluor 594 hydrazide (red, 250 µM) and Fluo-4 (green, 1 mM) from pipettes. Dye intensities normalized to values 20 min after whole-cell was plotted against time in one varicosity. Insets: green, Fluo-4; red, Alexa Fluor 594 in the same varicosity 20 and 70 min later. ***Ab***, Resting [Ca^2+^] for all cells calculated from Equations 1, 2 plotted over time after whole-cell access. Green circles from ***Aa***. ***B***, Ca^2+^ transients measured with low affinity dye. ***Ba***, Whole-cell-evoked action potentials evoked Ca^2+^ transients measured by line scans (***Bb***) through varicosities filled with Alexa Fluor 594 hydrazide and Fluo-5F. Line scan through the red bar in inset (***Bc***) showing intensities of five sequential line scans from the analyzed region in ***Bb***. Colors indicate time after whole-cell access. ***Bd***, Responses as ΔF/F + 1 (F = pre-stimulus Fluo-5F fluorescence). Ca^2+^ transients are invariant. ***Be***, Color-coded representation of line scan as ΔF/F + 1 from analyzed region in ***Bb*** after background subtraction. ***Bf***, Single Ca^2+^ transient 37 min after whole cell. Data fit by a single exponential. From fits, decay rates and peak responses were calculated. ***Ca***, In varicosities filled with Alexa Fluor 594 hydrazide and higher affinity Fluo-4 (1 mM), Ca^2+^ transients were evoked and Fluo-4 line scanned. The first recording (20 min after whole cell) was made as dye diffused into the varicosity (decay rate, τ= 0.53 s, ***Cb*** pink); 25 min later the amplitude was reduced and τ increased (τ = 1.7 s, red). ***Cc***, In other varicosities, at higher dye concentrations reduced ROI imaging (5 Hz) minimized bleaching. Transient intensity was plotted versus time and fitted with single exponentials to calculate τ and peak amplitude. Inset – varicosity imaged 80 min after whole-cell access at time points indicated by numbers. ***Da***, Comparison of effect of Fluo-4 and Fluo-5F on transients. Values of τ from fits to plotted versus time after whole-cell access. Data from each varicosity with Fluo-4 is linked with lines. The line through the Fluo-5F data are a least squares fit. ***Db***, Similar comparison of peak amplitudes. Values of τ and peak in Fluo-5F are constant those with Fluo-4 are time dependent.

To further illustrate co-diffusion of the two dyes, Alexa Fluor 594 hydrazide and Fluo-4 fluorescence (Fig. 2*Aa*) were normalized to their values when the first varicosity image (inset 1) was obtained (20 min after whole cell). For 70 min (until Fig. 2*Aa*, inset 2), fluorescence ratios between the dyes remained constant. Resting [Ca^2+^]_i_ was calculated from these data and the F_max_ of Fluo-4 fluorescence (obtained by repetitive stimulation), applied to Equation 3 (Materials and Methods). [Ca^2+^]_i_ remained stable for >1 h as dye concentrations rose (mean resting [Ca^2+^]_i_ = 81 ± 5 nM; 11 cells; Fig. 2*Ab*). At 80 min, Fluo-4 fluorescence increased more rapidly than Alexa Fluor 594’s revealing an increase in resting [Ca^2+^]_i_ (Fig. 2*Aa*, green circles). Therefore, we did not sample time points later than 80 min.

### Dependency of the Ca^2+^ signal on dye buffering capacity (κ_dye_)

Single CA1 axon varicosities loaded with low-affinity Ca^2+^-sensitive dye (200 µM Fluo-5F pipette concentration, *K*_d_ = 1.49 µM; *n* = 30) were imaged by line scanning (500 Hz) single action potential evoked Ca^2+^ transients (Fig. 2*B*). Responses were evoked by single action potentials through the recording pipette (Fig. 2*Ba*) during rising dye concentrations (Fig. 2*Bb*,*Bc*). Normalized as (ΔF/F + 1), these Ca^2+^ transients were invariant in amplitude and τ throughout the experiment (Fig. 2*Bd*), and the fluorescence transient was uniform across the varicosity within 6 ms of the stimulus (Fig. 2*Be*). Means of transients from sequential stimuli were plotted, and single exponentials fit to decays (Fig. 2*Bf*). From these fits, in all cells, transients from different varicosities at different axon locations gave reproducible amplitudes and values of decay time constant [τ; mean Δ[Ca^2+^]_i_ = 677 ± 10 nM (Materials and Methods; Eq. 3), mean τ = 119 ± 1.4 ms, *n* = 30].

Similar experiments were performed substituting the high affinity Ca^2+^ dye (Fluo-4, *K*_d_, 0.44 µM; 1 mM pipette concentration). Transient peak amplitudes reduced, and decay time constants (τ) increased as dye concentrations rose (Fig. 2*C*). This is consistent with Ca^2+^ buffering by Fluo-4, because the amplitude represents a proportion of the dye that is Ca^2+^-bound. As dye concentrations rise a smaller dye fraction binds Ca^2+^ entering. Unbound dye competes for Ca^2+^ with endogenous buffers, consequently, τ increases as rebinding to dye becomes more likely in cells (Neher and Augustine, 1992) or nerve terminals (Koester and Sakmann, 2000; Jackson and Redman, 2003; Brenowitz and Regehr, 2007). Indeed, as dye concentrations rose during the experiment, decay times became long enough that line scanning was not necessary. Axon terminals were instead imaged with sequential frames in two dimensions (Fig. 2*Cc*). When values of τ rose above 1 s, this imaging was used because it limited bleaching to repeat exposures.

An alternative explanation for increased τ is diffusional loss of endogenous buffers during whole-cell recording (Müller et al., 2007). However, this would occur regardless of dye concentration or affinity, be dependent on recording duration, and be accompanied by increased peak amplitudes. These effects did not occur (Fig. 2*D*). Indeed, during extended recording times, we compared Ca^2+^ transient τ's and peak amplitudes from Fluo-4 (*n* = 11 neurons) versus Fluo-5F (*n* = 30 neurons). As dye concentrations rose, the value of τ recorded with Fluo-4 increased, but in contrast the value obtained with Fluo-5F remained almost constant (Fig. 2*Da*). Similarly, the peak amplitude of the response measured as ΔF/F + 1, decreased over time when measured with Fluo-4 but again remained constant when recorded with Fluo-5F (Fig. 2*Db*). Thus, it is increasing dye buffering capacity (κ_dye_) not loss of endogenous buffer that causes the changes in peak ΔF/F and τ. Calculated dye concentrations combined with calibrated dye properties (Fig. 1) and Ca^2+^ transient amplitudes were therefore used to relate dye buffering to varicosity Ca^2+^ transients.

### Use of dyes to calculate Ca^2+^ entry and buffering

**Figure 3.**
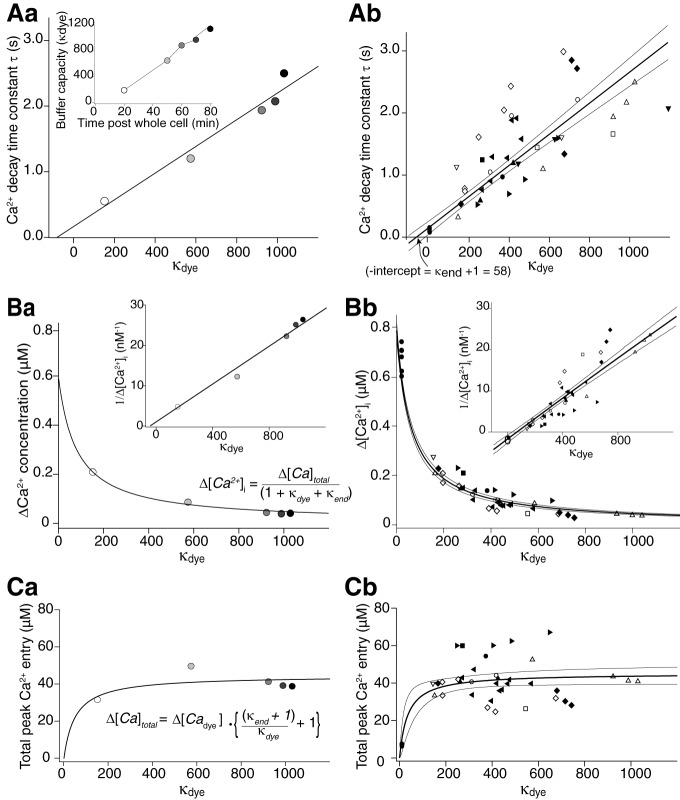
Analysis of Ca^2+^ transients and buffering using Ca^2+^-sensitive dyes. ***A***, From the fits to Ca^2+^ transients obtained with Fluo-4 and Fluo-5F at various values of κ_dye_, decay time constants (τ) were plotted against κ_dye_. ***Aa***, Example neuron. Inset, Increase in κ_dye_ over period of recording. ***Ab***, Recordings from all neurons (each symbol used represents a different cell). The negative intercept on the abscissa is a measure of endogenous buffering capacity (κ_end_) of the terminal (from Eq. 5). ***B***, Evoked change in [Ca^2+^]_i_ calculated from Equation 3 is plotted against dye buffering capacity (κ_dye_) for the same data. The curved line represents a fit of Equation 9. The inset represents the equivalent linear fit to the inverse of Δ[Ca^2+^]_i_ where the convergence of the fit with the abscissa represents κ_end_ and the slope is a function of Δ[Ca^2+^]_total_. The fits give values for the peak free Ca^2+^ concentration, the varicosity κ_end_ and total Ca^2+^ entering. ***Ba***, Example neuron. ***Bb***, Recordings from all neurons. ***C***, Values of total peak [Ca^2+^] bound to dye calculated from the product of measured Δ[Ca^2+^] and the κ_dye_ were plotted against κ_dye_. These data were fit to Equation 10. As the curve reaches an asymptote at high values of κ_dye_, this allows calculation of the total molar quantity of Ca^2+^ entering the varicosity following one action potential. ***Ca***, Example neuron. ***Cb***, Recordings from all neurons. Error bands are for 90% confidence intervals.

We determined κ_dye_ (Eq. 4) for each Ca^2+^ transient from varicosity dye concentrations, changes in varicosity-free Ca^2+^ (Δ[Ca^2+^]_i_) calculated from this data, and Equation 3. This value was plotted against time after obtaining whole-cell access for one example (Fig. 3*Aa*, inset). In 18 neurons (seven Fluo-5F, 11 Fluo-4) a linear fit to τ vs κ_dye_ (single cell example in Fig. 3*Aa*; data from all cells, Fig. 3*Ab*) gave an x intercept of –58 ± 40 and an estimate of the endogenous buffering capacity of the varicosity (κ_end_) of 57 (Eq. 5).

A plot of Δ[Ca^2+^]_i_ (Eq. 3) against κ_dye_ (Fig. 3*Ba* single example; data from all cells, Fig. 3*Bb*) demonstrated the relationship between κ_dye_ and the Ca^2+^ transient. A fit of Equation 9 to this data, or a linear fit to 1/Δ[Ca^2+^]_i_ vs κ_dye_ (Fig. 3*B*, insets), gives a peak free change in Ca^2+^ concentration (Δ[Ca^2+^]_I_) in the absence of dye throughout the terminal of 0.76 ± 0.03 µM. This is obtained from the *y*-axis intercept of the fit where κ_dye_ = 0. Constants from the same fit give total Ca^2+^ entry of 58 ± 6 µM and κ_end_ of 75 ± 6.

Total dye bound Ca^2+^ ([*Ca_dye_*]) was calculated for each transient either from the product of [Ca^2+^]_I_ and κ_dye,_ or from proportions of dye bound to Ca^2+^ calculated from the Hill equation. These gave values that differed by <5%. Data in Figure 3*C* are from the former (single cell Fig. 3*Ca*; combined data, Fig. 3*Cb*). If total Ca^2+^ entering each varicosity were constant for each action potential, then these data are represented by Equation 10, in which the asymptotic value of total Ca^2+^ entering the varicosity was 45 ± 3 µM. From this and each measured varicosity dimensions (volume mean = 1.7 ± 0.3 µm^3^, median = 1.2 µm^3^, *n* = 18) we determined total molar Ca^2+^ entering the varicosity (Table 1). κ_end_ is taken from fits of Equation 9 to Δ[Ca^2+^]_i_ (Fig. 3*B*). Peak [Ca^2+^]_i_ is for Ca^2+^ throughout the varicosity and total [Ca^2+^] entering is from fits to Equation 10 of data in Figure 3*C*. Results with the least errors were used and summarized (Table 1).

**Table 1.** Parameters of the varicosity and Ca^2+^ signaling

κ_end_	75 ± 10
Resting [Ca^2+^] (nM)	81 ± 5
Peak Δ[Ca^2+^]_i_ (nM)	760 ± 30
Total calcium entering (Δ[Ca]_total_; µM)	45 ± 3
Total molar Ca^2+^ entry (mol)	8.0 × 10^–20^
Median varicosity volume (µm^3^)	1.2 ± 0.3
Median varicosity surface area (µm^2^)	7.6 ± 1.0
Fluo-5F transient decay rate τ (ms)	119.5 ± 1.4
Calculated τ with 0 [dye] (s)	0.11 ± 0.09
Extrusion pump rate	7.6 × 10^6^ ± 1.1 × 10^6^ × (total #Ca^2+^ ions) M^–1^ s^−1^

### Ca^2+^ source and removal from the terminal

**Figure 4.**
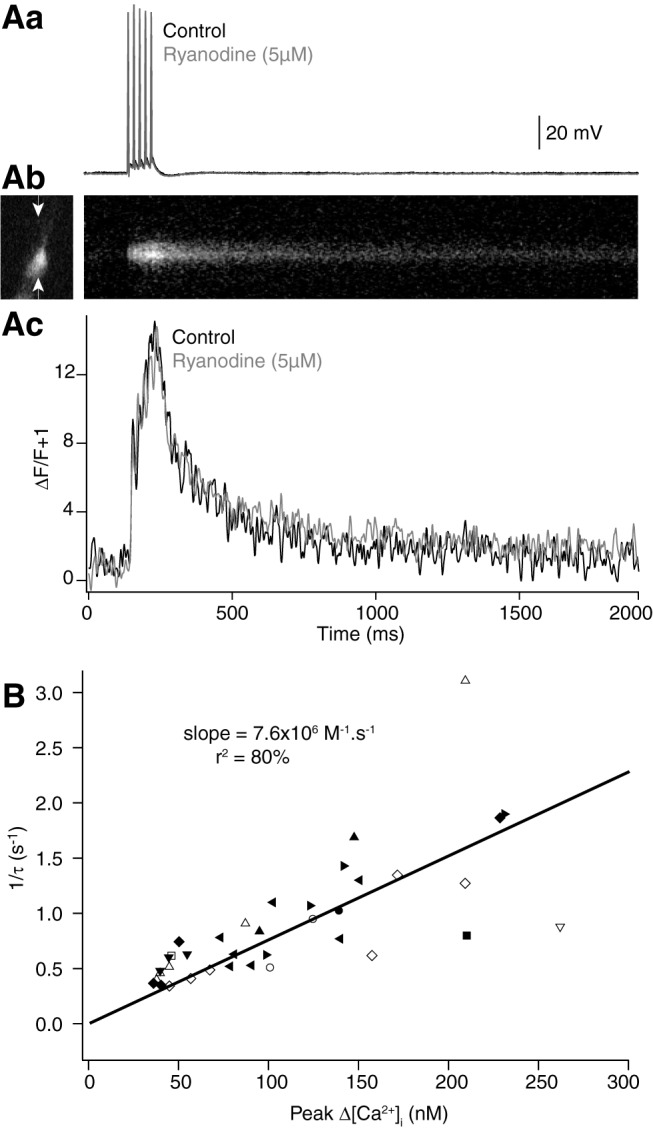
The Ca^2+^ transient is unaffected by block of release of Ca^2+^ from internal stores, and its peak amplitude is correlated with the inverse of its decay. ***A***, Repetitive stimulation evokes summating Ca^2+^ transients that are unaffected by ryanodine. ***Aa***, Five action potentials at 20 Hz evokes a summating Ca^2+^ transient measured using Fluo-5F in a CA1 neuron varicosity (***Ab***), which was unaffected by a 30-min application of ryanodine (5 µM; ***Ac***, gray). ***B***, Inverse of the Ca^2+^ transient decay rate versus peak Δ[Ca^2+^]_i_ for varicosities in which the value of κ_dye_ exceeded 150. From the slope, the extrusion rate of Ca^2+^ from the varicosity was determined (equal to slope × total molar Ca^2+^ entry). Each symbol used represent a different cell.

Internal stores might contribute to Ca^2+^ transients (Cochilla and Alford, 1998; Emptage et al., 2001; Scott and Rusakov, 2008). Calculations of Ca^2+^ entering, and κ_end_ will be distorted if secondary Ca^2+^ sources exist. In recordings with Fluo-5F, ryanodine (5 µM; to block store release) was superfused, and five action potentials (50 Hz; Fig. 4*A*) evoked a response on which ryanodine had no effect (to 111 ± 12% of control, 95% confidence interval of 90–132%, *n* = 5). Thus, ryanodine does not meaningfully alter Ca^2+^ transients even during trains of stimuli.

If Ca^2+^ extrusion is mediated by pumps with linear rates versus peak [Ca^2+^]_I_, then as the transient varies with κ_dye_, removal rates can be calculated. As dye concentration increases, κ_dye_ dominates κ_end_, and peak [Ca^2+^]_i_ available to pumps is reduced. Removal will be inversely proportional to available [Ca^2+^]_i_. The slope of 1/τ of the transient against peak [Ca^2+^]_i_ allows calculation of Ca^2+^ removal. Thus, these data obtained with Fluo-4 were plotted (Fig. 4*B*), and the extrusion rate [7.6 × 10^6^ × (total #Ca^2+^ ions) M^− 1^ s^−1^] calculated from the slope. The linearity of these data also provide evidence that secondary Ca^2+^ sources do not contribute to the transient, at least at these values of κ_dye_. A summary of experimentally determined properties of the varicosity is given in Table 1.

### Modeling of Ca^2+^ transients in varicosities

**Figure 5.**
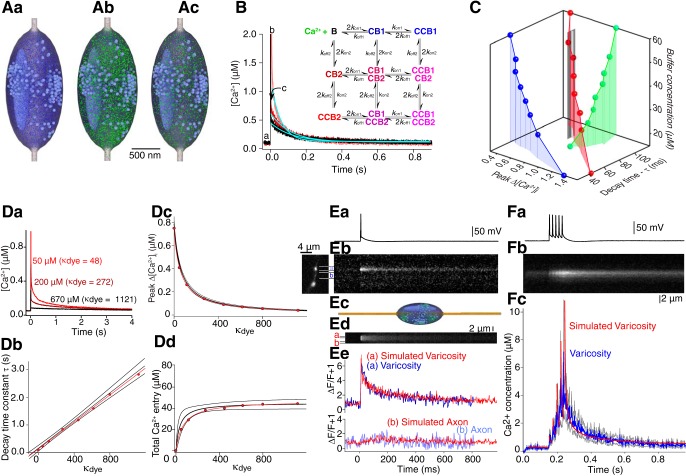
MCell simulation of Ca^2+^ transients and validation against experimental data. ***A***, The varicosity was modeled as an ellipsoid 2 × 1 × 1 µm and contained 320 synaptic vesicles, and one large internal structure to provide sites for Ca^2+^ intrusion (i.e., ER/mitchondrion). A variable quantity of Ca^2+^ buffer was modeled. Images show free Ca^2+^ ions (green) and Ca^2+^-bound states of calbindin_28K_ as per the color scheme in the kinetic model (***B***). Times indicated by letters in ***B***. Parameter from Table 1 and from varying buffer concentrations. Pre-stimulus state (***Aa***) at peak of stimulus (***Ab***) at first time point resolveable with fluorescence imaging (***Ac***). ***B***, Simulated total varicosity-free [Ca^2+^]_i_ following stimuli at three calbindin_28K_ concentrations (15 µM, pink; 40 µM, red; 60 µM, black). Single exponential fits (blue) were applied to these simulations and values of peak Δ[Ca^2+^]_i_ and decay (τ) were obtained from these fits. Inset shows kinetic scheme for the calbindin_28K_ 2:2 ratio. Parameters in Table 2. ***C***, 3D plot of simulated τ, peak ΔCa^2+^ against varying buffer concentrations (15–60 µM) following simulated varicosity stimulation. Blue, 3:1 ratio model of calbindin_28K_; red, 2:2 model of calbindin_28K_; green, model of calmodulin. Parameters in Table 2. The vertical black line and gray shading represent experimentally obtained τ, peak ΔCa^2+^ and their standard errors. These data converge only with the 2:2 ratio of calbindin_28K_ at a concentration of 39.7 µM. ***D***, Varying Ca^2+^-sensitive dye concentrations (Fluo-4; Table 1) were simulated and resultant Ca^2+^ transients graphed. ***Da***, Responses shown with dye concentrations of 50, 200, and 670 µM. ***Db–Dd***, Decay time, peak ΔCa^2+^, and total Ca^2+^ entry from these simulated transients over values of κ_dye_ from 0 to 1121 (red circles and lines) is plotted as per Figure 3*C* and overlaid on the fits and 90% confidence intervals of that experimental data. ***E***, Comparison to experimental data of simulation in axons. Action potentials (***Ea***) evoked varicosity Ca^2+^ transients (***Eb***) recorded with Fluo-5F that also allowed recording from axons. Such events were also simulated by calculating Ca^2+^ concentration along the axon and varicosity axis (***Ec***, yellow). ***Ed***, Simulated line scan. ***Ee***, top, Overlay of experimental ΔF/F + 1 from a varicosity (blue) and the simulated result (red). Bottom, Similar overlay but from adjacent axons. ***F***, Comparison to experimental data of simulations of trains of stimuli. ***Fa***, Five actions potentials at 50 Hz evoked Ca^2+^ transients recorded with Fluo-5F (***Fb***) quantified as Ca^2+^ concentrations (***Fc***, blue, gray lines 90% confidence interval from five varicosities). The same train was simulated (***Fc***, red). Simulation data fell within the 90% confidence interval of the data.

Imaging experiments provided resting [Ca^2+^]_i_, varicosity volumes, their endogenous buffering capacity, the peak free [Ca^2+^]_i_, the total Ca^2+^ entering, and its removal rate. The principal Ca^2+^ buffer in CA1 pyramidal somata is calbindin_28K_ and its concentration in somata and dendrites has been determined to be 40 µM (Müller et al., 2005), making it a candidate buffer in varicosities (Arszovszki et al., 2014) with well-characterized Ca^2+^-binding properties (Nägerl et al., 2000). Calmodulin with similarly well-characterized properties (Faas et al., 2011) has also been proposed as a dominant binding protein in these neurons. We constructed 3D models in the simulation environment MCell (Kerr et al., 2008; Tables 2, 3), to investigate Ca^2+^ entry, diffusion, buffering, and removal during action potential stimulation. Parameters and buffers (either calbindin_28k_ or calmodulin), used in this model were determined in this study, obtained from the literature, or varied to obtain best fits to the data (Table 2). A 3D mesh model was developed (Blender), from published data and from this study. Varicosities were represented by ellipsoids (Fig. 5*A*), 2 × 1 µm (volume = 1.2 × 10^−11^ l, the median measured varicosity volume) en passant to an axon (0.12 µm in diameter). They contained 320 40-nm diameter vesicles and an internal structure mimicking organelles (Fig. 5*A*). We modeled Ca^2+^ extrusion with a rate of 2.2 × 10^11^ M^− 1^ s^−1^ (from the number of Ca^2+^ ions entering and the slope from Fig. 4*B*) distributed to 2500 pumps over the plasma membrane and 1000 on the internal organelle mesh structure. A Ca^2+^ leak (1.77 × 10^5^ ions s^− 1^) similarly distributed achieved resting free [Ca^2+^]_i_ of 81 nM. Ca^2+^ buffering was modeled with three models of buffers (calbindin_28K_ 3:1 ratio, calbindin_28K_ 2:2 ratio or calmodulin). Calbindin_28K_ possesses four Ca^2+^-binding sites and models have been proposed for its Ca^2+^ binding (Nägerl et al., 2000) with 3:1 or 2:2 ratios of high-affinity and medium-affinity non-cooperative sites. The former model was slightly but not significantly favored in data fits *in vitro*, but the latter fit experimental data significantly better in a model of cerebellar Purkinje neurons (Schmidt et al., 2012) in which a correction to the on-rate accounted for intracellular Mg^2+^ (rates indicated in Table 2). Ca^2+^-calmodulin binding was also modeled using published parameters (Faas et al., 2011; Table 2). In our simulations, we used the same Mg^2+^ on-rate correction in our models for calbindin_28K_ and calmodulin. Evoked Ca^2+^ entry was modeled as a total of 45 µM Ca^2+^ entering the terminal in 2 ms (Table 1)

**Table 2.** Parameters used in the MCell model

Parameter	**Value**	Source of value
Resting free [Ca^2+^]_i_ (nM)	81	Eq. 3
Peak free Δ[Ca^2+^]_i_ (nM)	760	Eq. 9; Fig. 3*B*
Total [calcium] entering (Δ[Ca_total_]; µM)	45	Eq. 10; Fig 3*C*
Absolute extrusion rate Leak intrusion rate	2.2 × 10^11^ M^–1^ s^−1^ 1.77 × 10^5^ s^−1^	Fig. 4*B* × total # Ca^2+^ ions for resting [Ca^2+^]_I_ = 81 nM
Decay rate at 0 [dye] (τ)	0.110 s	Fig. 3*A*
Volume of model terminal (L) Cytosolic volume (L)	1.22 × 10^–11^ 1.07 × 10^–11^	Cytosolic volume excludes organelle and vesicle structures in the model
	**Dye parameters**	
Fluo-4 *K*_d_/*k*_on_/*k*_off_ F_min/_F_max_	0.44 µM/5 × 10^8^ M^–1^ s^−1^/221.5 s^−1^/750 s^−1^ 0.0232	Fig. 1
Fluo-5F *K*_d_/*k*_on_/*k*_off_	1.49 µM/5 × 10^8^ M^–1^ s^−1^/750 s^−1^	Fig. 1
	**Calcium buffers**	
Calbindin28K concentration Ratio of high to low affinity sites 3:1 ratio high affinity *k*_on1_/*k*_off1_ 3:1 ratio med. affinity *k*_on2_/*k*_off2_ 2:2 ratio high affinity *k*_on1_/*k*_off1_ 2:2 ratio low affinity *k*_on2_/*k*_off2_	15–60 µM 2:2 or 1:3 6.5 × 10^6^ M^–1^ s^−1^/2.405 s^−1^ 3.85 × 10^7^ M^–1^ s^−1^/44.44 s^−1^ 5.5 × 10^6^ M^–1^ s^−1^/2.6 s^−1^ 4.35 × 10^7^ M^–1^ s^−1^/35.8 s^−1^	Varied in model Varied in model (Nägerl et al., 2000; Schmidt et al., 2012)
Calmodulin concentration N-terminal *k*_on/off_ T N-terminal *k*_on/off_ R C-terminal *k*_on/off_ T C-terminal *k*_on/off_ R	15 – 60 µM 7.7 × 10^8^ M^–1^ s^−1^/1.6 × 10^5^ s^−1^ 3.2 × 10^10^ M^–1^ s^−1^/2.2 × 10^4^ s^−1^ 8.4 × 10^7^ M^–1^ s^−1^/2.6 × 10^3^ s^−1^ 2.5 × 10^7^ M^–1^ s^−1^/6.5 s^−1^	Varied in model Faas et al. (2011; stoichiometry is present with each EF hand lobe showing two sequential on and off rates represented by T and R)
Syt1 C2A *k*_on_/*k*_off_ Syt1 C2B *k*_on_/*k*_off_	2 × 10^8^ M^–1^ s^−1^/1.2 × 10^4^ s^−1^ 2 × 10^8^ M^–1^ s^−1^/8 × 10^3^ s^−1^ 2 × 10^8^ M^–1^ s^−1^/2 × 10^5^ s^−1^ 2 × 10^8^ M^–1^ s^−1^/4 × 10^4^ s^−1^ 2 × 10^8^ M^–1^ s^−1^/4 × 10^4^ s^−1^	Radhakrishnan et al. (2009)
	**Diffusion constants**	
D_Ca_	223 µm^2^ s^−1^	Allbritton et al. (1992)
D_Fluo-4_	75 µm^2^ s^−1^	Kong et al. (2013)
D_Fluo-5F_	75 µm^2^ s^−1^	Kong et al. (2013)
D_Calbindin_	0.2 fraction immobile	Schmidt et al. (2012)
	0.8 fraction mobile, 20 µm^2^ s^−1^	Schmidt et al. (2012)

**Table 3.** MCell model-specific parameters

Iteration interval (s)	4 × 10^–8^ (calbindin/calmodulin buffer determination)5 × 10^–9^ (syt1 and local Ca^2+^)
Partition size (µm; this is an MCell function that divides the volume into subvolumes to optimize calculations)	0.2
Molecular interaction radius (nm; radius at which two molecules may interact)	10
Microscopic reversibility (function that increase accuracy of reactions)	On

Buffer concentrations were first estimated by solving the Hill equation for values of total Ca^2+^ entry, and resting, and stimulated peak free [Ca^2+^]_i_. This assumes equilibrium at peak, and was expected to underestimate true buffer concentrations because Ca^2+^ transients are too rapid for equilibration. This gave 92.8 µM for calmodulin and 24.8 µM calbindin_28K_ (2:2 ratio of high-affinity and medium-affinity sites), or 19.7 µM (3:1 ratio).

To determine buffer parameters, simulated endogenous buffer concentrations were varied starting at steady-state results to compare simulations to experimentally determined peak free Δ[Ca^2+^]_i_ and τ (Fig. 5*B*; parameters in Tables 2, 3). Transient decays were fit with single exponentials omitting the first 4 ms of the simulation to avoid initial Ca^2+^ inhomogeneities. From these fits, peak free Δ[Ca^2+^]_i_ and τ were plotted against simulated buffer concentrations (Fig. 5*C*). Intersection of simulated and experimental values for τ and Δ[Ca^2+^]_i_ implies that model parameters are accurate and provides an estimate for buffer concentration and type. Models of calbindin_28K_ with a 2:2 binding ratio converge to within the 95% confidence interval of the experimental data (Fig. 5*C*, red line and markers). A least squares fit for this convergence predicts a calbindin_28K_ 2:2 concentration of 39.7 µM, in close agreement to 40–45 µM calbindin_28K_ obtained experimentally for rat CA1 pyramidal neuron somata (Müller et al., 2005). Neither the calbindin_28K_ 3:1 ratio nor calmodulin converged (Fig. 5*C*). Note, in cerebellar Purkinje neurons, models for calbindin_28K_ also favor 2:2 ratios (Schmidt et al., 2012). While other Ca^2+^ buffers are present, we conclude that simulating calbindin_28K_ with a 2:2 ratio of binding site has validity.

### Model validation of experimental results in a small terminal

We used simulations to determine if our experimental approach derived from (Neher and Augustine, 1992) is valid for small terminals. Effects of Fluo-4 (0–670 µM) were simulated and κ_dye_ calculated as for experimental data. As for experimental data, rising values of simulated κ_dye_ reduced peak [Ca^2+^]_i_ and increased τ. Peak [Ca^2+^]_i_ and τ were measured from single exponentials fitted to simulated data (Fig. 5*Da*) ommitting the initial 4 ms. Results were plotted with experimental data as τ versus κ_dye_ (Fig. 5*Db*), peak [Ca^2+^]_i_ versus κ_dye_ (Fig. 5*Dc*), and total Ca^2+^ entry captured by the dye vs κ_dye_ (Fig. 5*Dd*). In all cases, simulations fell within the 90% confidence limits of experimental data supporting the use of this approach in small varicosities and providing a validation of the simulation by simulating an uncontrolled variable.

Extrapolations of τ and Δ[Ca^2+^]_i_ (Fig. 3), to a hypothetical zero dye concentration where κ_dye_ = 0 (Fig. 3), compared with results for recordings using Fluo-5F shows a close match indicating that the dye did not significantly buffer the Ca^2+^ transient. Therefore, we used Fluo-5F to investigate Ca^2+^ signaling in detail and to further validate simulations with experimental data. In four preparations the orientation of the axon and varicosity allowed simultaneous line scanning of both, to compare the relative amplitudes of their Ca^2+^ transients. In axons farther than 1 µm from the varicosity no Ca^2+^ transient was observed (Fig. 5*Eb*,*Ee*, blue), indicating that Ca^2+^ does not escape from varicosities by diffusion. An equivalent simulation was performed, in which Ca^2+^-Fluo-5F binding was simulated in discrete volumes along the model axis (Fig. 5*Ec*, yellow), and ΔF/F + 1 calculated from simulated bound/unbound ratio of Fluo-5F. Results (mean, 20 random seeds) were plotted with overlaid experimental data (Fig. 5*Ee*, red). The simulated transient, like the experimental was confined to the varicosity with negligible signal seen 1 µm from the varicosity (Fig. 5*Ee*, lower traces).

**Figure 6.**
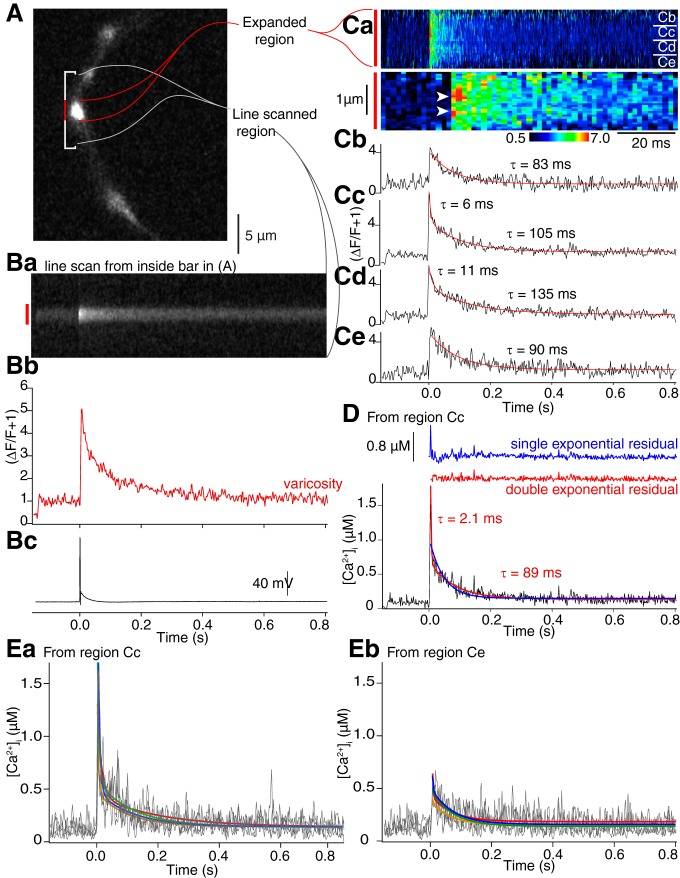
Ca^2+^ or Ca^2+^ dye complex can show rapid diffusion from presynaptic terminal hotspots immediately after the stimulus. ***A***, Axon varicosity containing both Alexa Fluor 594 hydrazide and Fluo-5F imaged using the Alexa Fluor dye. Bracketed region encompasses a single varicosity (center, red) and axon on either side (white) used for line scanning. ***B***, Mean of four line scans from region in ***A***. The varicosity exhibited a Ca^2+^ transient similar to those recorded in earlier figures (***Ba***). Mean intensity of the line scan through the varicosity (***Bb***, red bar), which showed a large increase in Ca^2+^ dye fluorescence following a single action potential (***Bc***). ***C***, The mean of four line scans within the varicosity (red in ***B***) displayed as ΔF/F + 1 (***Ca***, upper panel time scale as in graphs in ***D***, ***E*** below). Immediately after the stimulus there is a rapidly decaying component of the transient. The lower panel of ***Ca*** shows the two hotspots (arrowheads) at a 10× time scale. Data were then analyzed in quadrants (***Cb–Ce***). Center quadrants (***Cc***, ***Cd***). The center quadrants were well fit with double exponentials, the outer with single exponentials. ***D***, Data in ***Cc*** replotted to demonstrate the amplitude of free [Ca^2+^]_i_ within this region of the varicosity. As with the data in ***Cd***, this required a double exponential fit (single exponential and residual in blue, double exponential and residual in red). ***E***, Ca^2+^ transients (gray) were plotted for each of the four sequential responses averaged in ***C***. ***Ea***, Plotted from the region labeled (***Cc***); and (***Eb***) from the region labeled (***Ce***) in ***Ca***. Double exponentials were fit to these data for each of the four traces (colored traces).

We also tested simulations by comparing their results to train-evoked Ca^2+^ transients in Fluo-5F (200 µM)-labeled varicosities. Ca^2+^ transients were recorded during 5 action potentials (50 Hz; *n* = 5 cells; Fig. 6*Ca*). This caused a summating Ca^2+^ transient (Fig. 5*F*). Absolute Ca^2+^ concentrations were calculated and expressed as the mean of all responses (Fig. 5*Fc*, blue; seven responses) bounded by the 90% confidence interval (gray). Ca^2+^ transients simulated using MCell (five stimuli at 50 Hz; 10 seeds; Fig. 5*Fc*; red) fall within the 90% confidence intervals of the experimental data for the cell shown and for all five neurons.

### Spatiotemporal distribution of Ca^2+^ entry to the terminal

Calculations of varicosity [Ca^2+^] and its buffering assume that Ca^2+^ rapidly reaches spatial uniformity within varicosities. The overlap between single exponential fits to experimental data and fits to simulations (Fig. 5*D* indicates this approach is valid to calculate total Ca^2+^ entry and its buffering; however, in five neurons recorded with sufficiently high resolution, we observed repeatable, but non-uniform, Ca^2+^ distributions immediately after stimulation; Fig 6).

In each varicosity (Fig. 6*A*), line scans (mean of four transients in each of the five terminals; ΔF/F + 1 vs time; Fig. 6*B*), reveal brighter regions immediately post- stimulus (Fig. 6*Ca*; hotspots arrowed at faster time base, lower panel). However, these spots are close to the resolution limit of the microscope, and their intensity might have been affected by errors in background or prestimulus intensity. If hotspots represent localized Ca^2+^ entry then a faster local decay, that will not be altered by these errors, would represent diffusion from this site (mean τ overall = 119.5 ± 1.4 ms). Therefore, fits to exponentials were analyzed in line scan subregions. In the case illustrated, a single exponential well-fit Ca^2+^ transients away from hotspots (Fig. 6*Cb*,*Ce*), but did not adequately fit regions at hotspots. The hotspots were well-fit by double exponentials (τs of 6–11 and 105–135 ms; Fig. 6*Cc*,*Cd*). Similar results were obtained in all five neurons (mean τ_1_ = 9.0 ± 2.9 ms; τ_2_ = 124.5 ± 19.1 ms; sum of squares of residuals were significantly different between single and double exponential fits at the hotspots, *t*_(4)_ = 3.25, *p* = 0.015, but were not significantly different away from hotspots, *t*_(4)_ = 1.42, *p* = 0.11; the ratio of amplitudes of the first and second exponentials at the hotspots = 0.92. This was significantly higher than values obtained from double exponentials fit to data away from hotspots = 0.47, *t*_(4)_ = 3.82, *p* = 0.009). To illustrate the peak [Ca^2+^]_i_ recorded by Fluo-5F, experimental data are replotted (Fig. 6*D*, black) as [Ca^2+^]_I_ versus time, and is well-fit with a double exponential (red; residual above also in red, fast τ = 2.1 ms, peak [Ca^2+^]_i_ of 1.8 µM; vs 0.8 µM for the rest of the varicosity). In all five neurons, the mean peak free [Ca^2+^]_i_ = 2.7 ± 0.57 µM and mean fast τ = 3.3 ± 1.3 ms. By comparison, the data were poorly fit by a single exponential (blue, residual above, goodness of fit was again determined by comparing sums of squares of residuals. The sum of squares of these residuals were significantly different between single and double exponential fits at hotspots; *t*_(4)_ = 3.25, *p* = 0.016, but not away from hotspots, *t*_(4)_ = 2.00, *p* = 0.060).

These Ca^2+^ hotspots are repeatable. Examples from two locations (arrowed Fig. 6*C*) with an early fast Ca^2+^ transient (Fig. 6*Ea*) or lacking one (Fig. 6*Eb*) were analyzed in four sequential stimuli (five cells). As for the values obtained from values of ΔF/F at hotspots or non-hotspot regions, each [Ca^2+^]_i_ response was fit with a double exponential. Fast exponential amplitudes for the two regions were significantly different (the fast exponential amplitude at hotspots, 592 ± 170 nM and slow, 331 ± 68 nM, whereas at non-hotspots these amplitudes were 230 ± 141 nM and 303 ± 48 nM, *p* = 0.0052, two-factor ANOVA, respectively), but there was no significant difference between slower exponential amplitudes (*p* = 0.08).

### Simulation of localized Ca^2+^ entry

**Figure 7.**
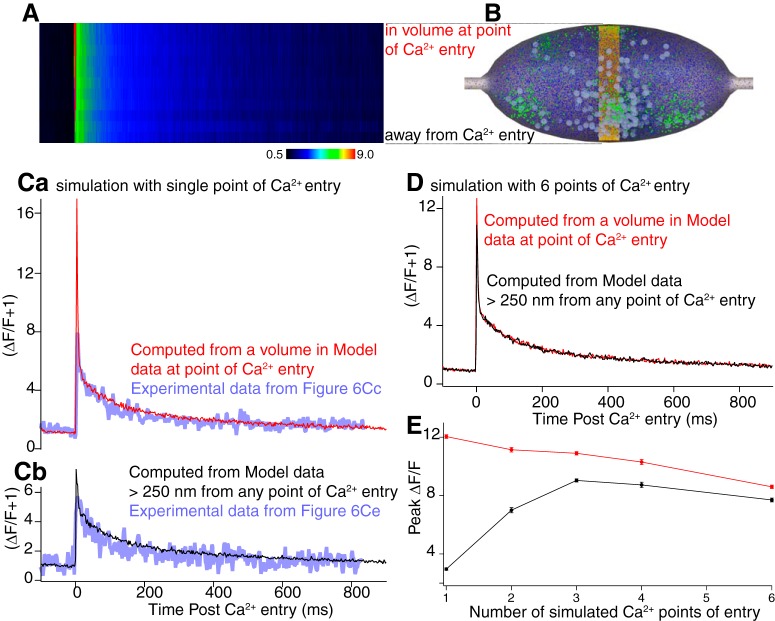
Simulations of Fluo-5F transients and clustering of Ca^2+^ entry. Line scans (***A***) of Fluo-5F transients were simulated by determining its simulated Ca^2+^ binding in volumes (yellow boxes) across the varicosity (***B***). ***Ca***, Simulated Fluo-5F ΔF/F + 1 transient (red) in the volume closest to Ca^2+^ entry (top of yellow band). Experimental data from Fig. 6*Cc*, blue. ***Cb***, Simulated Fluo-5F ΔF/F + 1 transient in the volume at the bottom of the yellow band in ***B*** opposite the site of Ca^2+^ entry (black). Experimental data from Fig. 6*Ce* overlaid in blue. ***D***, Ca^2+^ entry was simulated at six sites, including one placed at the plasmalemma, at the top of the yellow band in ***B***. The Fluo-5F transient is shown from this site (red) and away from sites of Ca^2+^ entry from the bottom of the yellow band in ***B*** (black). ***E***, Graph of peak amplitude of double exponential fit to the simulated transient at a site of Ca^2+^ entry (red) and away from these sites (black) in varicosities where Ca^2+^ entry was at one to six sites.

We determined whether discrete placement of Ca^2+^ entry within the simulation could reproduce the experimental non-uniform Ca^2+^ distribution. In simulations, Ca^2+^ entry was located at one to six plasma membrane sites. However, in all cases, the total Ca^2+^ entry summed to 45 µM (Table 2) over the whole varicosity. Experimental line scanning was simulated (20 random seeds) by simulating values of Fluo-5F ΔF/F + 1 (Fig. 7*A*) in a line of discrete volumes across the model varicosity (Fig. 7*B*, vertical yellow band). One end of this band always included only one Ca^2+^ entry site. ΔF/F + 1 values, were resampled to rates obtained during experimental line scanning (500 Hz) to create a simulated line scan matrix (Fig. 7*A*) equivalent to the experimental data.

Simulation results were plotted from the two ends of the yellow band, obtained when all Ca^2+^ entry (45 µM) was at one point (Fig. 7*B*, top, point of simulated Ca^2+^ entry; bottom, away from Ca^2+^ entry; Fig. 7*C*, overlaid with experimental data, blue). There is a substantial difference between amplitudes of the earliest peak at the Ca^2+^ entry site (Fig. 7*Ca*, red) compared to the other side of the simulated varicosity (Fig. 7*Cb*, black). Similar simulation results were plotted where Ca^2+^ was evenly distributed at six locations, one of which was at the same location as above. Little difference in peak amplitude was seen at the Ca^2+^ entry site and away from it (Fig. 7*D*).

Double exponentials were fit to the simulations. τ’s of fast exponentials were within the 90% confidence limits of fits of early components of experimental data (simulations from 3 to 4 ms; experimental data; Fig. 6*D*, 3.3 ± 1.3 ms). Peak amplitudes of simulated Fluo-5F ΔF/F early components were obtained for fits to all distributions of Ca^2+^ entry at the site of entry and across the varicosity at the opposite end of the yellow band (Fig. 7*B*) from this site. Substantial differences in peak amplitudes at a point of Ca^2+^ entry compared to the opposite side of the varicosity away from Ca^2+^ entry, were observed only when Ca^2+^ entry was at one or two sites (that is when at least 50% of total varicosity Ca^2+^ entry was localized to one site; Fig. 7*E*). Thus, to obtain experimental local peaks in Ca^2+^ (Fig. 7), clustering of VGCCs may occur.

### Proximity of the point of Ca^2+^ entry to the release machinery and paired-pulse facilitation

**Figure 8.**
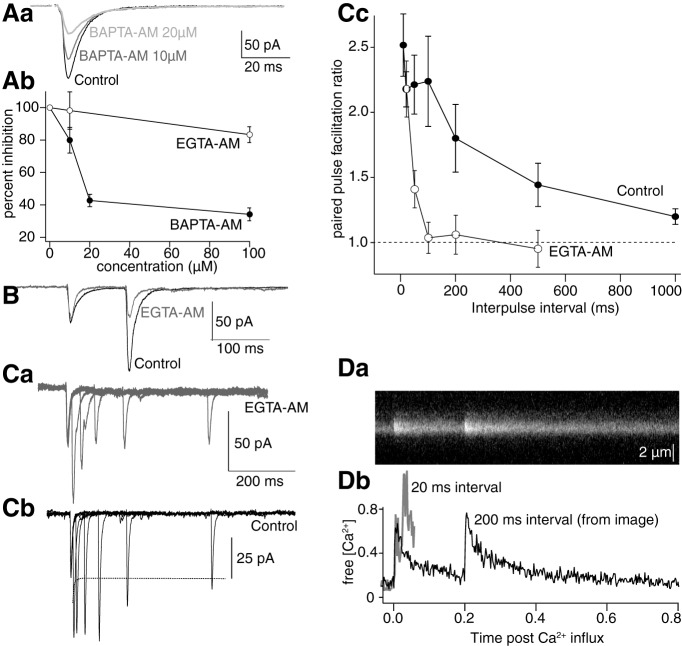
Paired-pulse potentiation is not explained by presynaptic residual Ca^2+^ concentration. ***A***, EPSCs (in AP5 and bicuculline to block NMDAR and GABA_A_R responses) recorded in subicular pyramidal neurons following stimulation of CA1 pyramidal axons. Effects on EPSC amplitudes of BAPTA-AM or EGTA-AM were recorded after 15–20 min of perfusion. ***Aa***, BAPTA-AM (10–100 µM) caused dose-dependent inhibition of the EPSC (graphed in ***Ab***). EGTA-AM (10–100 µM) had little effect. ***B***, Paired-pulse responses were obtained at 10 Hz (six neurons) EGTA-AM (10 µM) was added to the superfusate (gray). Paired-pulse facilitation was prevented. ***Ca***, In three neurons with EGTA-AM (10 µM), the interstimulus interval was varied from 20 to 500 ms. Paired pulse potentiation was prevented over intervals >50 ms. By contrast control paired-pulse potentiation was seen in all neurons tested over the same range of stimulus intervals (***Cb***). ***Cc***, Graph showing paired pulse potentiation for these intervals in control (filled circles) and after superfusion of EGTA-AM (open circles). The dashed line represents the first stimulus amplitude. Errors expressed as SEM between preparations. ***D***, Ca^2+^ transients were stimulated in paired pulses. ***Da***, Line scan image of stimuli at 200-ms intervals. ***Db***, Paired-pulse responses at 20-ms (gray) and 200-ms (black) intervals.

The proximity of Ca^2+^ entry to its molecular targets that cause vesicle fusion can be estimated by comparing effects of BAPTA (rapidly binds Ca^2+^) to EGTA (slower binding; Adler et al., 1991) on synaptic transmission. To record synaptic responses from CA1 synapses, their axons were stimulated (1/15 or 1/30 Hz; Hamid et al., 2014). Whole-cell recordings were made from their target subicular pyramidal neurons and EPSCs recorded (in bicuculline, 5 µM; AP5, 50 µM) to isolate AMPA receptor responses. BAPTA-AM was superfused (10–100 µM, 15–20 min) and reduced EPSC amplitudes dose dependently (to 34 ± 4% at 100 µM, *n* = 13; Fig. 8*A*). In contrast, EGTA-AM (20–100 µM was significantly less effective (100 µM, *n* = 12, to 83 ± 5% of control; Fig. 8*A*,*B*; *t*_(22)_ = 8.0, *p* = 2.6 × 10^−8^). These results imply a close spatial association (tens of nanometers; Adler et al., 1991) between VGCCs and syt1 responsible for exocytosis.

While Ca^2+^ evoked neurotransmitter release thus requires close coupling of Ca^2+^ channels to the Ca^2+^ target, repetitive stimulation evokes synaptic facilitation (Zucker and Regehr, 2002). This facilitation is thought to be caused by residual Ca^2+^, Ca^2+^-buffer saturation (Klingauf and Neher, 1997; Matveev et al., 2004), or Ca^2+^-dependent processes distinct from the fast exocytic machinery (Fioravante and Regehr, 2011), for example, by acting at syt7 (Jackman et al., 2016; Jackman and Regehr, 2017). At CA1 pyramidal cell presynaptic terminals we show that this facilitation is Ca^2+^ dependent. In similar recordings to Figure 8*A*, paired pulse stimuli were presented to CA1 pyramidal axons with interpulse intervals of 100 ms (Fig. 8*B*). This gave a mean facilitation ratio of 1.85 ± 0.191 (amplitude of EPSC 2/EPSC 1, *n* = 5, *t*_(4)_ = 2.4, *p* = 0.03). The paired pulse enhancement was abolished by EGTA-AM (20 µM; facilitation ratio after EGTA-AM = 1.0 ± 0.1, *t*_(4)_ = 0.4, *p* = 0.35).

We then determined time-courses of paired-pulse facilitation with EPSCs in subicular pyramidal neurons (Fig. 8*C*). In three of the preparations treated with EGTA-AM (20 µM) the paired-pulse interval was varied (20–500 ms; Fig. 8*Ca*). In EGTA-AM, facilitation was abolished at intervals >50 ms. In contrast in controls, facilitation was recorded at intervals from 20 to 500 ms (Fig. 8*Cb*). Facilitation ratios from controls (*n* = 7) and after EGTA-AM (*n* = 3) were plotted from 20- to 1000-ms intervals (approximate duration of varicosity Ca^2+^ transients; Fig. 8*Cc*). In contrast to this facilitation of postsynaptic responses, presynaptic Ca^2+^ transients did not show augmentation at 20- or 200-ms interpulse intervals (although at 20 ms the responses summed) and showed no summation at 200-ms intervals. Ca^2+^ transients were recorded and evoked following whole-cell recording as for Figure 2*B*, except that paired pulses of action potentials were evoked at 20- or 200-ms intervals. Amplitudes of the paired evoked Ca^2+^ transients (Δ[Ca^2+^]_i_) were not significantly altered (Fig. 8*D*; at 20-ms intervals 2nd response was 115 ± 19% of 1st, *n* = 7, *t*_(6)_ = 0.98, *p* = 0.18; at 200-ms intervals 2nd response was 100 ± 10% of the 1st, *n* = 3, *t*_(2)_ = 0.03, *p* = 0.49). Thus, while paired pulse facilitation is Ca^2+^ dependent, as indicated by its sensitivity to EGTA, Ca^2+^ transients do not measurably augment at the scale of the entire terminal to cause this potentiation.

### Simulating paired-pulse presynaptic Ca^2+^ transients

**Figure 9.**
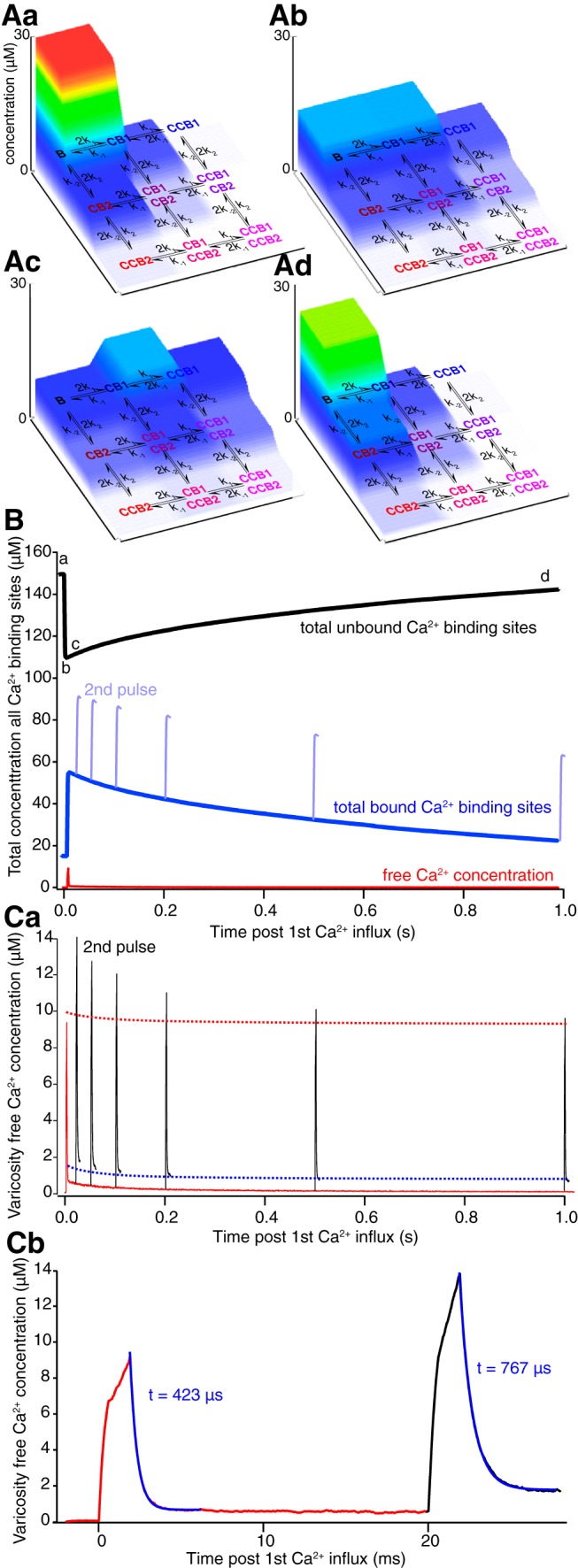
Simulations of Ca^2+^ buffering in the varicosity. ***A***, Using simulation parameters previously determined, the kinetics of calbindin_28K_ binding were simulated and displayed as state diagrams from before a simulated stimulus (***Aa***) as well as during and after the stimulus (***Ab***, ***Ac***, ***Ad***) from time points indicated in ***B***. Concentrations of calbindin_28K_ in each of its possible Ca^2+^-bound or unbound states throughout the varicosity is plotted on the vertical axis and color coded to concentration. The horizontal axes display Ca^2+^ binding to high-affinity (B1) and medium-affinity (B2) sites (C, single Ca^2+^ bound; CC, two Ca^2+^ bound). ***B***, Graph of total concentration of vacant Ca^2+^-binding sites on calbindin_28K_ throughout the varicosity (black) and total bound Ca^2+^ (blue) after a stimulus and 2nd pulse in light blue at varying interpulse intervals. Free Ca^2+^ concentration is also shown (red) to the same scale for a single pulse. ***Ca***, Graph of free Ca^2+^ concentration after a single stimulus (red) throughout the varicosity and after 2nd stimuli at varying paired-pulse intervals (black). Red dashed line indicates theoretical value of linear summation of a second pulse. Blue dashed line represents the linear sum of the Ca^2+^ signal that is resolvable using imaging at 500 Hz. ***Cb***, Expansion of the time course of paired pulses from ***Ca*** for pulses at 50 Hz. Color scheme as for ***Ca***. Single exponential fits to the fast component of decay of the Ca^2+^ transients and their decay times (τ) are shown in blue.

To address experimental limitations of analyzing Ca^2+^ at the spatiotemporal resolutions of the vesicle fusion machinery and its activation, we simulated Ca^2+^, Ca^2+^ buffer states, and effects of repetitive stimulation on unbound and bound Ca^2+^ (Fig. 9) using parameters previously determined. Within the simulation, at rest, >90% of calbindin_28K_ is unbound (Fig. 9*Aa*,*B*). Stimulation causes partial occupancy of all calbindin_28k_ states (Fig. 9*A*). However, 2/3 of all bound states remain unoccupied even at peak occupancy (Fig. 9*Ab*,*B*). Nevertheless, unbinding is slow and full recovery takes longer than 1 s (Fig. 9*Ad*,*B*). We then determined the effect of paired pulses over intervals from 20 to 1000 ms. Although calbindin_28K_ was not saturated at any intervals (Fig. 9*B*, blue), the second pulse achieved higher peak free [Ca^2+^] than the first (Fig. 9*C*, difference between 2nd peaks, black, and red dashed line, that represents linear summation) and showed a slower decay (Fig. 9*Cb*; τ of exponential increased from 423 µs on the first stimulus to 767 on the second). This enhanced second peak, only resolvable by simulation is seen despite the fact that the Ca^2+^ signal recorded at the base of this initial transient resolvable with imaging is only enhanced by <200 nm and only at the very shortest intervals (linear summation of the component resolvable by experimental imaging is demonstrated by the blue dashed line). The increased amplitude and decay rate is driven by higher occupancy of calbindin_28K_ by Ca^2+^. We conclude that the transient Ca^2+^ signal resulting from diffusion of Ca^2+^ throughout the varicosity does show an enhanced amplitude due to partial Ca^2+^ buffer saturation. However, this signal is computed from a much larger volume than the Ca^2+^ transients within tens of nanometers of the VGCCs that evokes fusion by interacting with syt1.

### Activation of syt1 by evoked presynaptic Ca^2+^ entry

**Figure 10.**
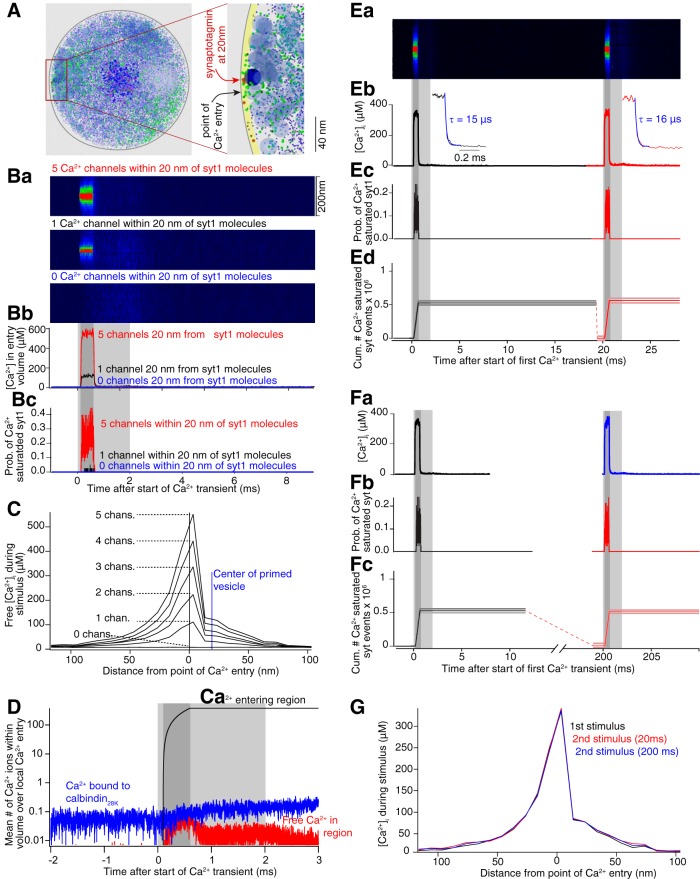
Paired pulses do not alter nanometer domains of Ca^2+^ entry. ***A***, Characteristics of Ca^2+^ entry, buffering, and dispersal analyzed close to Ca^2+^ entry modeled within 20 nm of a vesicle at the plasmalemma. Left panel, View along axon axis including varicosity contents (blue dots, calbindin_28K_; green, Ca^2+^, vesicles; light blue, organelle). Right panel, Magnified model showing volume at the membrane (yellow band, where simulated Ca^2+^ was determined) and vesicles (blue circles, one dark blue at Ca^2+^ entry site). The latter vesicle distorts the spatial symmetry of the Ca^2+^ signal (***C***, ***G***; Shahrezaei and Delaney, 2004). ***Ba***, Ca^2+^ transients expressed as a simulated line scan versus time from yellow band in ***A*** centered at one point of Ca^2+^ entry for five channels (red), one channel (black), and zero local channels (blue) active for 0.5 ms (dark gray) against a background of 45 µM entering the entire varicosity for 2 ms (light gray) in ***Bb***, where data are from the center 20 nm of ***Ba***. ***Bc***, Probability (from 100 simulations) of syt1 model showing occupancy of all five Ca^2+^-binding sites for five (red) and one (black) local Ca^2+^ channels. Gray shaded regions as for ***Bb***. ***C***, Spatial distribution of the Ca^2+^ transient peaks from the end of the dark gray shading in ***B*** for 0 through five channels at the local site of Ca^2+^ entry. ***D***, Total Ca^2+^ entering a volume within 20 nm of three channels (black), the free Ca^2+^ in this volume (red), and the Ca^2+^ in the volume bound to buffer. Loss of Ca^2+^ by diffusion is the difference between the black trace and the sum of the black and red traces (traces are on a logarithmic scale). Gray shaded regions as per ***B***. ***E***, Paired-pulse Ca^2+^ transients from yellow region in ***A*** simulating three local channels located within 20 nm of syt1. ***Ea***, Simulated line scans as for ***Ba***, but with a second pulse simulated 20 ms after the first. ***Eb***, Ca^2+^ transients within 20 nm of local Ca^2+^ entry repeated at a 20-ms interval. Insets show single exponential fits and decay rates (blue) of the paired pulse local Ca^2+^ transient. ***Ec***, Probability of syt1 binding five Ca^2+^ ions. ***Ed***, Cumulative number of syt1-five Ca^2+^-binding events to emphasize these are equal in both pulses. ***F***, Paired pulse results (***Fa–Fc***) as per ***Eb–Ed*** but at a paired-pulse interval of 200 ms. ***G***, Spatial distribution of the peak free Ca^2+^ entry in pulse 1 (black) and the second pulse at 20 ms (red) and 200 ms (blue) from volume in ***A***, ***B***.

Syt1, the Ca^2+^ sensor for exocytosis in CA1 pyramidal neurons, has two C2 domains with five Ca^2+^-binding sites, some of which have mM affinities for Ca^2+^ (Südhof and Rizo, 2011). This requires syt1 to be <100 nm from the Ca^2+^ source (Adler et al., 1991; Augustine et al., 1991) as demonstrated also here (Fig. 8). It is unclear whether all sites must bind Ca^2+^ to evoke exocytosis (Radhakrishnan et al., 2009), although there is evidence that very low affinity interactions at the C2A domain (Ubach et al., 1998) is important for syt1 interactions with syntaxin and for vesicle fusion. We simulated syt1 Ca^2+^ binding (for parameters, see Table 2) at the plasmalemma (Fig. 10*A*) using data that membrane interaction of the C2A domain enhances its Ca^2+^ binding (Radhakrishnan et al., 2009). Ca^2+^ entry and buffering were again simulated. To determine the requirements for Ca^2+^ entry in the immediate vicinity of syt1 for full binding to occur, simulations were performed with six Ca^2+^ entry sites across the surface of the varicosity such that a total of 45 µM entered at each stimulus. Syt1 molecules were placed at 20, 100, and 200 nm from one of these sites (Fig. 10*A*), adjacent to a vesicle. This proximal Ca^2+^ site varied from containing an equivalent of zero to five simulated channels (0.25 pA each, 0.5-ms open time).

More than 20 nm from the proximal Ca^2+^ site no full syt1 binding events were recorded (100 random seeded simulations). Thus, the peak free [Ca^2+^]_i_ throughout the varicosity (9.3 µM; Fig. 9*C*, red) is insufficient to occupy all five syt1-binding sites. However, even one simulated VGCC within 20 nm of syt1 allowed this binding (Fig. 10*Ba–Bc*, black) and more channels increased the probability of this binding (Fig. 10*Ba–Bc*, red). With five channels, there was a small probability of syt1 fully binding Ca^2+^ at five binding sites simultaneously when it was 100 nm from the Ca^2+^ source. Within 20 nm from the Ca^2+^ source, one channel raised the peak transient concentration to 110 µM; five channels to 560 µM (Fig. 10*Bb*,*Bc*). At this proximity full Ca^2+^ occupancy of all five syt1 Ca^2+^ sites occurred transiently throughout the stimulus.

The narrow spatial half-widths (Fig. 10*C*) and rapid decay of Ca^2+^ within tens of nm of VGCCs indicate rapid Ca^2+^ removal from these volumes. Plots of total Ca^2+^ entering the 20-nm scale region, Ca^2+^ bound to calbindin_28K_ within the region, and free Ca^2+^ are shown on a log scale (Fig. 10*D*) to encompass the range of concentrations when activation of three VGCCs was simulated at this site (similar results were obtained by clustering all Ca^2+^ entry at these points). The arithmetic difference between Ca^2+^ entry to this region, the Ca^2+^ source by simulating three open channels (Fig. 10*D*, black curve), and Ca^2+^ remaining in the region which is the sum of [Ca^2+^]_i_ (red trace) and Ca^2+^ bound to buffer (blue trace) is the Ca^2+^ that leaves the region by diffusion. This latter amount is 99.8% of the Ca^2+^ that enters the region indicating that at this spatial scale, removal of Ca^2+^ is dominated by diffusion. Thus, local Ca^2+^ concentrations (tens of nanometers from the channels) are also dominated by diffusion during paired pulse stimulation when Ca^2+^ buffers throughout the varicosity are not close to saturation (Fig. 9). Consequently, no significant paired-pulse facilitation (20- and 200-ms intervals) of the local Ca^2+^ signal (within 200 nm of VGCCs) and no change in the decay rate from the peak of the Ca^2+^ signal (Fig. 10*Eb*, insets) was seen at these scales. Similarly full syt1-Ca^2+^ binding at all five sites was the same for the first and second stimuli (Fig. 10*Ec*,*Ed*, 20-ms intervals; Fig. 10*Fb*,*Fc*, 200-ms intervals; Fig. 10*G*).

## Discussion

CA1 pyramidal neurons make en-passant synapses at subicular varicosities (Finch et al., 1983; Tamamaki and Nojyo, 1990). We show that evoked Ca^2+^ transients are reliably activated in these varicosities, regardless of distance from the stimulated soma. Using a low affinity dye that did not significantly buffer entering Ca^2+^ (Fluo-5F), Ca^2+^ transients recorded over >1 h showed consistent amplitudes and decay rates. This implies quantal fluctuation of neurotransmission is not mediated by fluctuations in total CA1 varicosity Ca^2+^ entry, although full Ca^2+^ occupancy of syt1 is very sensitive to local Ca^2+^ placement and we cannot distinguish total Ca^2+^ in the varicosity from that entering at active zones. The result is not broadly applicable across all neurons. Evoked Ca^2+^ entry in cerebellar granule cell varicosities vary (Brenowitz and Regehr, 2007), whereas responses in hippocampal dentate granule cells (Jackson and Redman, 2003), cortical pyramidal cells (Koester and Sakmann, 2000), and lamprey axons (Photowala et al., 2005) are reliable.

### Quantitation and simulation of Ca^2+^ entry and buffering

This reliability, alongside our ability to introduce known Ca^2+^ dye concentrations, allowed calculations of Ca^2+^ buffering capacity, molar Ca^2+^ entering, and extrusion rates. If we assume a VGCC current of 0.25 pA for 1 ms during action potentials (Weber et al., 2010), then on average 27 channels open per action potential per varicosity with a mean channel density of 7 µm^−2^. This is consistent with findings that either single channels evoke release (Stanley, 1993, 2015; Haydon et al., 1994; Bertram et al., 1996) or few channels are necessary (Bucurenciu et al., 2008, 2010), and allow efficient coupling of Ca^2+^ to exocytosis (Scimemi and Diamond, 2012). Interestingly channel clustering and distance to the fusion apparatus can vary, giving synaptic responses with different probabilities even within one Calyx (Fekete et al., 2019).

Calculated properties of the Ca^2+^ transient enabled creation of MCell simulations (Kerr et al., 2008) to investigate Ca^2+^ entry, diffusion, and buffering. Calbindin_28K_ dominates Ca^2+^ buffering in CA1 pyramidal neurons (Müller et al., 2005) and its binding properties are known (Nägerl et al., 2000) enabling its simulation. Experimentally determined peak [Ca^2+^]_i_ and τ were well-described by simulating calbindin_28K_ with a 2:2 ratio of high-affinity and medium-affinity Ca^2+^ sites. Remarkably, 3D plots of τ vs peak [Ca^2+^]_i_, vs calbindin_28K_ concentrations converge with experimental data at a calbindin_28K_ concentration (39.7 µM) identical to these neurons’ somata and dendrites (40 µM for somata; Müller et al., 2005). This 2:2 ratio of binding sites (Nägerl et al., 2000) also well fit data from cerebellar Purkinje neurons (Schmidt et al., 2012). Two other buffer configurations, a 3:1 ratio of sites for calbindin_28K_, or of calmodulin failed to converge with experimental data (Fig. 5). With the caveat that Ca^2+^ buffering utilizes a mix of buffers, our findings are consistent with calbindin_28k_ as the dominant buffer. The model also reproduced independent features of experimental data; responses to repetitive stimulation and a failure to detect Ca^2+^ diffusion from varicosities to axons. Simulations also validated use of dye as buffer within small varicosities. Simulating rising concentrations of Fluo-4 recapitulated experimental data showing effects of buffer on measured Ca^2+^ transient decays, peak amplitudes, and total Ca^2+^ entering varicosities (Fig. 5).

### Localization and clustering of Ca^2+^ entry

We compared experimental data and simulations to investigate presynaptic Ca^2+^. Presynaptic VGCCs localize to active zones (Khanna et al., 2007) and bind SNARE complex proteins (Mochida et al., 1996; Harkins et al., 2004; Szabo et al., 2006). Furthermore, release may be activated by single VGCCs (Stanley, 1993; Bertram et al., 1996; Shahrezaei et al., 2006), although, it remains unclear whether presynaptic Ca^2+^ entry occurs at channel clusters (Llinás et al., 1992b; Bertram et al., 1996; Shahrezaei and Delaney, 2005) or a uniform distribution of channels, where individual VGCCs associate with primed vesicles in a 1:1 ratio. However, because Ca^2+^ signals are smaller and faster than our imaging resolution, Ca^2+^ entering the terminal, even at discrete points, will appear closer to uniformity throughout the terminal. Additionally, dye-Ca^2+^-complex diffusion might smooth variations, although we have demonstrated that Fluo-5F (10–35 µM) caused little perturbation because most Ca^2+^ binds endogenous buffers rather than dye. (κ_dye_ = 10–20 for Fluo-5F vs 75 for κ_end_). Nevertheless, in recordings where signal-to-noise ratios were favorable, we recorded localized Ca^2+^ signaling. These regions show faster early τ’s, close to 3 ms (as fast as we can record). Peak free [Ca^2+^]_i_ within these regions reached 4 µM. While this does not represent concentrations causing exocytosis (Adler et al., 1991; Augustine et al., 1991; von Gersdorff and Matthews, 1994; Schneggenburger and Neher, 2000), it indicates non-uniform VGCC distributions, and channel clustering. We used MCell simulations of various VGCC distributions to explain localized Ca^2+^ transients. Substantial spatial variation was only seen in simulated Fluo-5F responses if half or more of the channels in model synapses were clustered at one site.

### Summation of Ca^2+^ transients and short-term plasticity

These synapses show paired-pulse facilitation that, as in other synapses (Katz and Miledi, 1967; Kamiya and Zucker, 1994; Zucker and Regehr, 2002), is Ca^2+^ dependent (Kamiya and Zucker, 1994; Mukhamedyarov et al., 2009) and follows the time course of presynaptic residual Ca^2+^. However, although peak evoked Ca^2+^ transients of 4 µM, and simulated concentrations of 10 µM throughout the varicosity may sum, Ca^2+^ concentrations local to syt1 exceed 100 µM. This suggests that summating Ca^2+^ throughout the varicosity cannot modify paired-pulse responses by acting directly at syt1. Alternatively Ca^2+^, during stimulus trains, might saturate endogenous buffers (Neher, 1998) to evoke larger transients (Jackson and Redman, 2003), or subsequent Ca^2+^ entry might be enhanced (Müller et al., 2008). In CA1 varicosities, experimentally applied paired pulse stimuli at 20- or 200-ms intervals reveal no significant alteration of the 2nd Ca^2+^ transient amplitude. Later responses in stimulus trains of five stimuli do show non-linear summation, perhaps attributable to buffer saturation (Neher, 1998) or secondary Ca^2+^ sources (Cochilla and Alford, 1998; Llano et al., 2000; Emptage et al., 2001; Scott and Rusakov, 2006), although notably, we recorded no effect of ryanodine at a dose that unloads Ca^2+^ stores (Alford et al., 1993).

To investigate Ca^2+^ at spatiotemporal scales relevant to molecular interactions, simulation was used. Our Ca^2+^-dye buffering data, supported by simulations, demonstrate that most Ca^2+^ entering the varicosity is buffered endogenously at imaging time scales, similar to results from other synapses (Koester and Sakmann, 2000; Jackson and Redman, 2003). Simulations also indicate that endogenous buffers re-release Ca^2+^ over seconds, and subsequent stimuli force occupancy of most Ca^2+^ buffer binding sites. Supralinear rises in imaged and simulated Ca^2+^ transients after more than two stimuli are due to buffer saturation. Simulation data indicate that total free Ca^2+^ concentrations averaged throughout the varicosity reach 9.3 µM after a single stimulus, and that buffer saturation indeed allows whole-terminal, paired-pulse enhancement of millisecond scale Ca^2+^ transients up to 1 s after the first stimulus (Fig. 9). However, this does not account for Ca^2+^ at scales of tens of nanometers and picoseconds in which Ca^2+^ binds to syt1.

Simulation at these nanometer scales shows Ca^2+^ dispersal from them is clearly dominated by diffusion and consequently there was no detectable difference in the amplitudes or distribution of two transients in paired pulses at intervals of 20 or 200 ms at this scale. Indeed, modeling binding of five Ca^2+^ ions to syt1 indicates that Ca^2+^ entry within <50 nm causes its full occupancy. This result, also indicates clustering of Ca^2+^ channels may contribute to release at this synapse. Indeed, clusters of channels that are constrained to be further than 30 nm from the fusion apparatus has been proposed in calyceal synapses (Keller et al., 2015), although in those synapses Ca^2+^ requirements for release are as low as 25 µM (Schneggenburger and Neher, 2000). However, full Ca^2+^ occupancy of the syt1 model is not enhanced by paired pulses. If residual Ca^2+^ is responsible for paired pulse facilitation (Zucker and Regehr, 2002), then these data point to a Ca^2+^-binding site distinct from syt1, such as syt7 (Jackman et al., 2016; Jackman and Regehr, 2017). This effect might alternatively be consistent with an effect of residual bound Ca^2+^ on vesicle priming (Neher and Sakaba, 2008) and the size of the readily releasable pool (Thanawala and Regehr, 2013).

We conclude that the Ca^2+^ responsible for evoked release at CA1 neuron varicosities reaches hundreds of micromolar at small clusters of Ca^2+^ channels local to the release machinery. These clusters may represent up to half of the Ca^2+^ entry in the terminal for which fewer than 30 Ca^2+^ channels open. As at most synapses, the Ca^2+^ channels must be within tens of nanometers of the fusogenic Ca^2+^ sensors, and simulations of Ca^2+^ indicate that diffusion dominates its dispersal at this scale. Thus, neither paired pulse Ca^2+^ accumulation (Wu and Saggau, 1994), nor buffer saturation (Neher, 1998; Matveev et al., 2004), nor Ca^2+^ pumps substantially impact Ca^2+^ binding to syt1. Nevertheless, buffer saturation during repetitive stimulation causes a varicosity wide enhancement of Ca^2+^ transient amplitudes which may impact short-term enhancement by recruiting other Ca^2+^-binding proteins.

## Synthesis

Reviewing Editor: Christian Hansel, University of Chicago Decisions are customarily a result of the Reviewing Editor and the peer reviewers coming together and discussing their recommendations until a consensus is reached. When revisions are invited, a fact-based synthesis statement explaining their decision and outlining what is needed to prepare a revision will be listed below. Note: If this manuscript was transferred from JNeurosci and a decision was made to accept the manuscript without peer review, a brief statement to this effect will instead be what is listed below.

The authors have appropriately responded to all comments raised by the reviewers at Journal of Neuroscience.
